# Identification of Archaea-specific chemotaxis proteins which interact with the flagellar apparatus

**DOI:** 10.1186/1471-2180-9-56

**Published:** 2009-03-16

**Authors:** Matthias Schlesner, Arthur Miller, Stefan Streif, Wilfried F Staudinger, Judith Müller, Beatrix Scheffer, Frank Siedler, Dieter Oesterhelt

**Affiliations:** 1 Department of Membrane Biochemistry, Max Planck Institute of Biochemistry, Am Klopferspitz 18, 82152 Martinsried, Germany; 2 Molecular Network Analysis Group, Max Planck Institute for Dynamics of Complex Technical Systems, Sandtorstrasse 1, 39106 Magdeburg, Germany; 3 Institute of Microbiology, Technische Universität Braunschweig, Spielmannstraße 7, 38106 Braunschweig, Germany

## Abstract

**Background:**

Archaea share with bacteria the ability to bias their movement towards more favorable locations, a process known as taxis. Two molecular systems drive this process: the motility apparatus and the chemotaxis signal transduction system. The first consists of the flagellum, the flagellar motor, and its switch, which allows cells to reverse the rotation of flagella. The second targets the flagellar motor switch in order to modulate the switching frequency in response to external stimuli. While the signal transduction system is conserved throughout archaea and bacteria, the archaeal flagellar apparatus is different from the bacterial one. The proteins constituting the flagellar motor and its switch in archaea have not yet been identified, and the connection between the bacterial-like chemotaxis signal transduction system and the archaeal motility apparatus is unknown.

**Results:**

Using protein-protein interaction analysis, we have identified three proteins in *Halobacterium salinarum *that interact with the chemotaxis (Che) proteins CheY, CheD, and CheC2, as well as the flagella accessory (Fla) proteins FlaCE and FlaD. Two of the proteins belong to the protein family DUF439, the third is a HEAT_PBS family protein. In-frame deletion strains for all three proteins were generated and analyzed as follows: a) photophobic responses were measured by a computer-based cell tracking system b) flagellar rotational bias was determined by dark-field microscopy, and c) chemotactic behavior was analyzed by a swarm plate assay. Strains deleted for the HEAT_PBS protein or one of the DUF439 proteins proved unable to switch the direction of flagellar rotation. In these mutants, flagella rotate only clockwise, resulting in exclusively forward swimming cells that are unable to respond to tactic signals. Deletion of the second DUF439 protein had only minimal effects. HEAT_PBS proteins could be identified in the chemotaxis gene regions of all motile haloarchaea sequenced so far, but not in those of other archaeal species. Genes coding for DUF439 proteins, however, were found to be integral parts of chemotaxis gene regions across the archaeal domain, and they were not detected in other genomic context.

**Conclusion:**

Altogether, these results demonstrate that, in the archaeal domain, previously unrecognized archaea-specific Che proteins are essential for relaying taxis signaling to the flagellar apparatus.

## Background

Many archaeal and bacterial species have the ability to sense environmental parameters and use this information to direct their movement in response to gradients towards more favorable locations [1]. This process, called taxis, is in both prokaryotic domains of life based on a modified two-component signal transduction system ([2-5], reviewed in [6]), and a motility organelle. The best understood motility organelle in bacteria, and the only one known in archaea, is the flagellum, a rotating, propeller-like structure (reviewed for example in [7-9]. Pili have been observed on the surface of many archaeal species, but their cellular function is unknown [10]). In response to external stimuli, the taxis signal transduction system modulates the frequency by which the flagellar motor changes its direction of rotation, and thus enables a biased random walk, and leads to movement to places with improved environmental conditions (reviewed in [11]).

Even though several variations of the taxis signaling system exist in different bacterial and archaeal species (see for example [12]), the overall mechanism, as well as the proteins involved, are conserved (for review see [6]). The receptors, also known as methyl-accepting chemotaxis proteins (MCP), sense a multitude of environmental stimuli such as various chemicals, oxygen, osmolarity and, in *H. salinarum*, also light. They regulate the autophosphorylation activity of the histidine kinase CheA, which is coupled to them by the adaptor protein CheW [13-15]. After autophosphorylation, the phosphoryl group is transferred from CheA to the response regulator CheY [16]. Phosphorylated CheY (CheY-P) is the flagellar motor switch factor [4,17]. Hence CheA acts as an integrator of diverse stimuli to generate an unambiguous output for the flagellar motor. Other proteins mediate adaptation to the signal (CheR, CheB, CheC, CheD, CheV) [18-23] and removal of the phosphate from CheY-P (CheZ, CheX, CheC, FliY) [16,24,25].

In bacteria, CheY-P binds to the flagellar motor switch protein FliM [26], which forms together with FliN and FliG, and in *B. subtilis *also FliY, the motor switch complex. The binding site of CheY-P is the highly conserved N-terminal region of FliM [27]. Without bound CheY-P, the flagellar motor in bacteria rotates in one default direction. Binding of CheY-P increases the probability that the motor switches to rotation in the opposite direction (reviewed in [28]).

The taxis signal transduction system of *H. salinarum *is built from 18 receptors (called halobacterial transducer proteins, Htrs), and the Che proteins A, Y, W1, W2, R, B, C1, C2, C3, and D [29,30]. Due to its ability to perform phototaxis, *H. salinarum *is an excellent model organism for studying cellular responses. In several studies, detailed data of the halobacterial response to light has been obtained [31-33], which allowed the generation of a quantitative model of the flagellar motor switch and its sensory control in this organism [34,35]. However, in spite of the good understanding of the switch cycle in *H. salinarum *on a systems level, the underlying molecular mechanisms remain unclear.

In archaea and bacteria, the flagellar apparatus is composed of three functional units: the flagellar filament, the motor, and its switch, which allows the motor to change its rotational direction. Despite this superficial similarity, these motility organelles are distinct structures in both domains, which are not related to each other (see [8,9,36] for review). For the proteins constituting the bacterial flagellar apparatus, no homologs have been detected in archaeal genomes, suggesting very strongly that the archaeal motility apparatus must be built from different components [8]. Furthermore, the archaeal flagellar motor is not driven by proton-motive force (PMF) like most bacterial motors, but either by ATP directly or by an ATP-dependent ion gradient which is not coupled to PMF (except via the H^+^-ATP synthase) [37]. In some respects, archaeal flagella resemble bacterial type IV pili more than bacterial flagella [38,39]. Known components of the archaeal flagellar apparatus are the flagellins, which compose the filament, and a number of conserved proteins that are coded by genes located close to the flagellin genes in archaeal genomes: the flagella accessory genes *flaC*, *flaD*, *flaE*, *flaF*, *flaG*, *flaH*, *flaI*, and *flaJ *[40,41]. In *H. salinarum *and other archaea of the families Halobacteriales and Methanomicrobia, the FlaC and FlaE proteins are fused to one polypeptide [42]. The exact role of the Fla proteins is not understood, but it has been shown by deletion mutations that they are required for flagellation [43,44]. A role in flagellar biosynthesis was suggested, because FlaI and FlaJ are homologous to proteins from the bacterial type II secretion system and type IV pili biogenesis system [8,43].

CheY-P is the flagellar motor switch factor also in *H. salinarum *and probably also other archaea [4,5]. However, the interaction site of CheY-P is unknown, since for its target protein in bacteria, FliM, just as for all other proteins constituting the bacterial flagellar apparatus, no homologs can be found in archaeal genomes [6,8,45]. No equivalent to the CheY-P binding peptide has been identified either.

Besides CheY-P, fumarate is a further factor involved in flagellar motor switching, both in archaea (*H. salinarum*, [46,47]) and bacteria (*E. coli *and *S. typhimurium*, [48]). In *E. coli*, fumarate reductase (FRD) was identified as the target of fumarate at the motor, where it was shown to interact with the flagellar motor switch protein FliG [49]. In *H. salinarum*, which has neither a FRD nor a FliG, fumarate must act by a different, till now unknown mechanism.

Hence, the connection between the archaeal flagellar apparatus, the bacterial-like taxis signal transduction system, and the enigmatic fumarate pathway has remained elusive. Here we report the identification of three proteins that interact with CheY, CheC2, and CheD as well as FlaCE and FlaD, thereby building a link between the taxis signal transduction system and the archaeal flagellar apparatus. By assaying deletion mutants, we demonstrated that two of these proteins are essential for control of the direction of rotation of the flagellar motor. Two of the proteins belong to the protein family DUF439. We found that the members of this family are generally and exclusively present in archaeal *che *gene regions. We conclude that DUF439 describes essential archaeal chemotaxis proteins for which we propose the name CheF.

## Results

### OE2401F, OE2402F, and OE2404R interact with Che and Fla proteins

**Figure 1:**
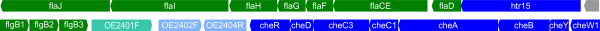
**Chemotaxis and motility gene cluster of H. salinarum**. Genes involved in chemotaxis are shown in blue and motility genes in green. The proteins investigated in this study are shown in light blue (the homologs OE2402F and OE2404R) and cyan. A protein of unknown function is colored gray.

Protein interaction analysis of the halobacterial Che proteins (Schlesner *et al*., unpublished; see Additional file 1 for details) revealed two proteins of unknown function, OE2402F and OE2404R, as interaction partners of CheY, CheD, and CheC2. These proteins are homologous to each other and are coded by adjacent genes, located between the *che *genes and the type B flagellins (Figure 1).

**Figure 2:**
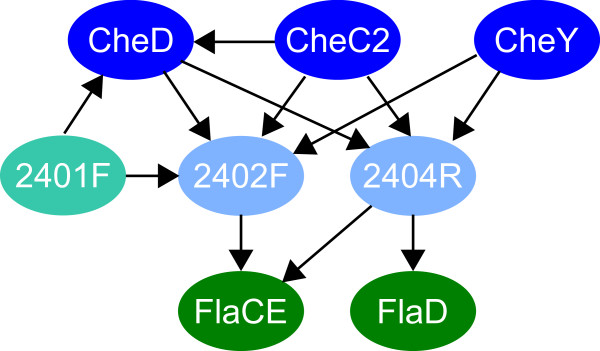
**Interactions of the newly identified proteins**. The arrows indicate the direction bait – prey in the pull-down experiments. See Additional file 1 for details.

To determine the role of OE2402F and OE2404R, these proteins were used as baits in additional bait fishing experiments. Both proteins were shown to interact with the flagellar accessory proteins FlaCE, and OE2404R also with FlaD (Figure 2; see Additional file 1 for details). The third protein, coded by a gene located between the *che *gene region and flagellins, OE2401F, was also subjected to protein interaction analysis, although it was not detected as an interaction partner in previous experiments. OE2401F was shown to interact with CheD and OE2402F.

These results indicate that all three proteins play a role in the chemotaxis signaling pathway of *H. salinarum*. Due to their interaction with Che proteins as well as with Fla proteins, the newly identified proteins build a link between the chemotaxis signal transduction system and the archaeal flagellar apparatus.

### Construction of in-frame deletion mutants

To elucidate the function of the newly identified proteins, in-frame deletion strains for OE2401F-OE2404R (referred to as Δ1, Δ2, and Δ4) and a double deletion ΔΔOE2402F OE2404R (Δ2–4) were created using a two-step recombination method [50]. As host, two *H. salinarum *strains were used: Strain R1 was used, because it is considered as wildtype and this strain was previously used for PPI analysis (Schlesner *et al*., unpublished; see Additional file 1 for details). The same deletion mutations were also constructed in strain S9, because S9 cells are better suited for motion analysis and determination of the flagellar rotational bias, whereas R1 cells tend to stick to the glass surface of the microscope slides [51]. Clones that had undergone the second recombination event were screened for the absence of the target gene(s) by PCR and confirmed by Southern blot analysis using probes for the target gene and its upstream region (see Additional file 2). DNA from the deletion strains did not hybridize with the gene probe, and showed the expected size decrease when probed with the gene's upstream region.

Since the deletions in both parent strains S9 and R1 exhibited the same phenotype, they will be discussed together in the following sections. As independent biological replicates, the use of two parent strains gives a high degree of certainty for the phenotypic findings.

### OE2401F and OE2402F are essential for chemotaxis and phototaxis

**Figure 3:**
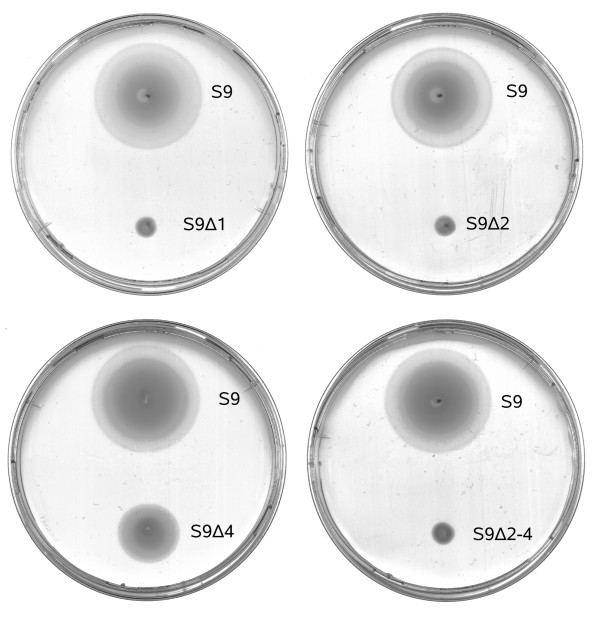
**Swarming ability of the deletion strains**. Representative swarm plate for each deletion in S9 after three days of growth at 37°C.

To examine the effect of the deletions on chemotaxis and motility, the deletion strains were analyzed by swarm plate assays. A swarm plate is a semi-solid agar plate in which the cells are inoculated. The agar concentration is low enough to allow movement of the cells in the agar. After point inoculation the cells grow, metabolize various nutrients, and create a concentration gradient. Cells which are motile and capable of chemotaxis move along this gradient away from the inoculation site, forming extended rings, called swarm rings. Figure 3 shows representative swarm plates for each deletion in S9, compared to wildtype (see Additional file 3 for all swarm plates). After three days of growth, the wild type strains formed large swarm rings. The deletion strains Δ1, Δ2, and Δ2–4 did not show any swarming. Δ4 cells produced swarm rings, but of a reduced size.

**Figure 4:**
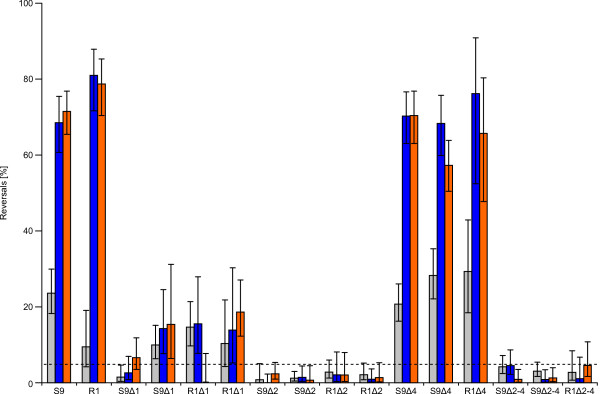
**Reversals of the wild type and deletion strains as measured by computer-based cell-tracking**. The percent reversal in a 4 second interval was determined either without stimulation (spontaneous, gray bar), after a blue light pulse (blue bar), or after a step down in orange light (orange bar). Error bars represent the 95% confidence interval. The dashed line indicates the estimated maximal tracking error of 5%. Two clones of each deletion strain were measured, except for R1Δ4 and R1Δ2–4.

Reduced or impaired ring formation on swarm plates can be due to defects in signal transduction or flagellar motility. In order to determine the defects of the deletion strains, their swimming ability was evaluated by microscopy, and the frequency of reversal of their swimming direction was measured with a computer-based cell-tracking system (Figure 4; see Additional file 4 for details). This system automatically determines the rate of reversing cells over a certain observation time [52].

Visual inspection clearly demonstrated that all deletion strains were motile without detectable swimming defects. The wild type strains showed in a 4 s observation interval a reversal rate of 10% (R1) and 25% (S9) in the unstimulated state. Upon stimulation with a blue light flash or orange light step down (both are repellent stimuli), wild type cells responded effectively with reversal rates of 70–80%.

In the strains Δ2 and Δ2–4, very low reversal rates of up to 5% were measured, both spontaneous and after stimulation. These strains displayed a smooth-swimming phenotype with hardly any switching. Similar results were obtained for the Δ1 strains. The reversal rates for three of the Δ1 clones were slightly higher than the estimated tracking error of 5%, but this may have been due to the low number of cells evaluated for these clones, which is also reflected by the broader confidence intervals. A significant increase of reversals after repellent stimulation could not be detected, indicating that this deletion has disabled the response to repellent stimuli. It leads to a strongly reduced switching frequency or even also to a smooth-swimming phenotype.

For Δ4, no significant difference was visible compared to wild type cells, either with or without photophobic stimulation.

### Δ1, Δ2, and the double deletion Δ2–4 show almost 100% CW rotational bias

To further characterize the defects of the deletion strains, the flagellar rotational bias was investigated by dark-field microscopy [53,54]. These measurements were taken only with the S9 strains and, except for Δ1, only one clone for each deletion was analyzed because the results were in complete agreement with the other phenotypic findings. The two S9Δ1 clones were investigated because they showed a slightly different phenotype in the phototaxis measurements (smooth-swimming vs. some residual switching).

The numbers of cells observed swimming forward (clockwise (CW) rotating flagella) and backward (counterclockwise (CCW) rotating flagella) are shown in Table 1. Wildtype cells showed a distribution between forward and backward swimming of close to 50:50, as expected [32,54]. Cells of the deletion strain Δ1, Δ2, and the double deletion Δ2–4, showed a bias toward forward swimming of almost 100%. The slight discrepancy of both S9Δ1 clones found in the cell tracking assay also showed up in this experiment, proving the reliability of the applied methods. Δ4 cells exhibited a rotational distribution of nearly 50:50, similar to wildtype.

**Table 1:** Flagellar rotational bias of the deletion mutants.

Strain	CW	CCW	CW (%)
S9	290	210	58
S9Δ1 C1	494	6	99
S9Δ1 C2	481	19	96
S9Δ2	500	0	100
S9Δ4	511	498	51
S9Δ2–4	499	1	100

The flagellar rotational direction was analyzed by dark-field microscopy. Cells with clockwise (CW) rotating flagella are pushed forward by their right-handed flagellar bundle, whereas cells with counterclockwise (CCW) rotating flagella are pulled backward [53]. The flagella and the direction of movement of the cell can be seen under the dark-field microscope and thus the rotational direction be determined. Shown is the number of cells in CW and CCW swimming mode at the time point of observation, as well as the percentage of CW swimming cells.

### The deletion phenotypes are not caused by inhibited expression of Che and Fla proteins

To rule out the possibility that the observed phenotypes are caused by altered *che *or *fla *gene expression levels, quantitative Realtime Reverse Transcription PCR (qRT-PCR) was applied (Table 2). The genes of the *che *operon as well as the *fla *genes CE, F, G, H, I, J are cotranscribed [43,55], so not all genes needed to be analyzed separately. *cheR *and *cheY *were chosen for analysis because *cheR *is at the border of the *che *operon, next to the deleted genes, and *cheY *was an additional control. *cheC2 *and *cheW2*, which are not located in the *che *operon, were not tested separately, because the deletion of these genes does not cause a smooth-swimming phenotype (unpublished observations). Additionally, *flaH *was chosen as a representative of the *fla *genes, although a defect in Fla protein expression seemed *a priori *unlikely since no motility defect was observed.

**Table 2:** Che and Fla protein expression in deletion strains.

Strain	Clone	CheR	CheY	FlaH
Δ1	1	1.24	1.06	1.76
	2	1.11	1.21	1.28
Δ2	1	1.58	-1.46	1.39
	2	1.00	-1.37	1.30
Δ4	1	1.24	1.08	2.03
	2	-1.05	1.46	1.65
Δ2–4	1	1.14	-1.16	1.77
	2	-1.96	-1.45	-1.37

The mRNA levels in the deletions in S9 were determined by qRT-PCR. Given is the fold difference in the mRNA level of the deletions compared to S9 wildtype. Each result is the average of two replicates.

The qRT-PCR curves were analyzed using the 2−ΔΔCt MathType@MTEF@5@5@+=feaagaart1ev2aaatCvAUfKttLearuWrP9MDH5MBPbIqV92AaeXatLxBI9gBaebbnrfifHhDYfgasaacPC6xNi=xH8viVGI8Gi=hEeeu0xXdbba9frFj0xb9qqpG0dXdb9aspeI8k8fiI+fsY=rqGqVepae9pg0db9vqaiVgFr0xfr=xfr=xc9adbaqaaeGaciGaaiaabeqaaeqabiWaaaGcbaGaeGOmaiZaaWbaaSqabeaacqGHsislcqqHuoarcqqHuoarcqWGdbWqdaWgaaadbaGaemiDaqhabeaaaaaaaa@335A@ method with normalization to the constitutively expressed *fdx *gene [56]. In none of the tested cases was a significant difference between deletion and wildtype observed.

### Complementation of the deletion strains reverted their phenotype to that of wildtype

All deletions in the S9 background were complemented by reintroducing the deleted gene *in cis*. The phenotype of the complementations was examined by swarm plates and, for the single deletions, by motion analysis. In these assays, all complementations behaved exactly like the wild-type strain (see Additional file 5), confirming that the phenotypes observed in the mutants were a direct result of their gene deletions.

### Bioinformatics analysis

To collect information on the three unknown proteins and to test if the findings obtained in *H. salinarum *are potentially transferable to other archaeal species, a bioinformatics analysis was done. The starting point was a homology search and querying databases like COG [57] and Pfam [58]. The goal was to identify orthologs from other organisms for which some knowledge might exist, and to unravel correlations between the occurrence of the here investigated proteins and Che and Fla proteins. For this, an extensive search for Che and Fla orthologs in all published archaeal genomes was performed (see Additional file 6).

OE2401F is classified as a HEAT_PBS or HEAT family protein [58]. These proteins are predicted to contain short bi-helical repeats. Beside the HEAT-like repeats, no other domain could be detected. In the COG database, OE2401F is identified as member of the FOG ("Fuzzy Orthologous Groups") COG1413, described to contain HEAT repeat proteins. This COG belongs to the functional category C, "Energy production and conversion". No functional information for this COG is given.

The repeats in the protein sequence of OE2401F led to a high number of non-significant matches in database searches. Thus it was not possible to identify a reliable set of orthologs from other organisms, and no conclusions about co-occurrence of this protein family with *che *or *fla *genes could be drawn. Close homologs were identified in the *che *and *fla *gene regions of the halophilic archaea *N. pharaonis *and *H. marismortui*. These homologs are, like in *H. salinarum*, adjacent to a DUF439 gene. Additionally, proteins with HEAT-like repeats are present in all sequenced haloarchaeal genomes (the above mentioned, *H. walsbyi*, and *H. salinarum*) in other genomic context. For none of these proteins could any functional knowledge be obtained.

#### Homologs of OE2402F and OE2404R are found generally and exclusively in archaeal che gene regions

OE2402F and OE2404R are annotated as conserved hypothetical proteins. They are homologous to each other and belong to the protein family DUF439 [58] and to the cluster of orthologous groups COG2469. DUF439 is described as "archaeal protein of unknown function", and COG2469 as "uncharacterized conserved protein".

**Figure 5:**
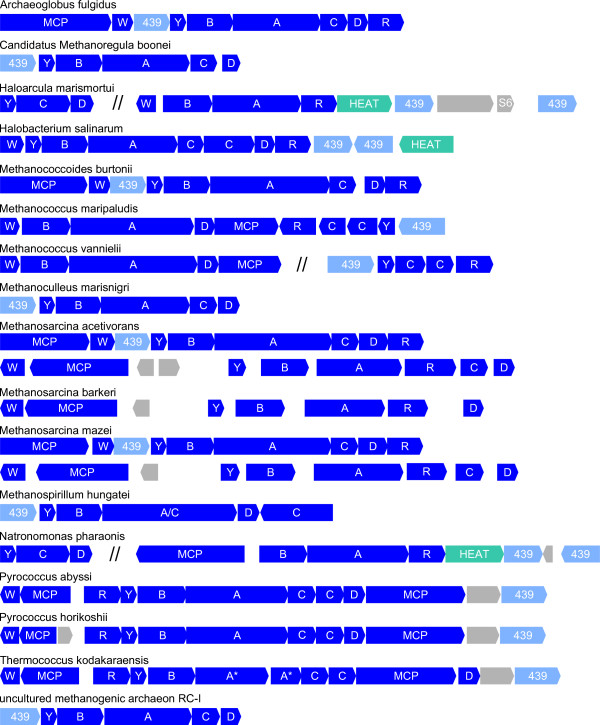
**Organization of chemotaxis genes in archaeal genomes**. Known chemotaxis genes (indicated by gene letter) and genes coding for receptors (Methyl-accepting chemotaxis proteins, MCP) are shown in blue. Genes coding for proteins of the family DUF439 are shown in light blue and genes coding for HEAT domain proteins in cyan. Gray indicates that, where no name is given, the function of the coded protein is unknown, or the protein is probably unrelated to chemotaxis (S6: 30S ribosomal protein S6e). A//sign indicates separated genome regions. The asterisk indicates that this protein is interrupted by a frame-shift mutation.

Homology searches showed that no members of the family DUF439 can be found outside the domain Archaea. Among the archaea, the presence of such a gene strictly correlated with the presence of *che *genes (see Additional file 6). The only exceptions were *Methanocaldococcus jannaschii*, which does not possess *che *genes but has a DUF439 homolog, and *Methanosarcina barkeri*, that has *che *genes but no DUF439. Examination of the genomic context revealed that the DUF439 genes are always located in the chemotaxis gene regions (Figure 5). The exceptions were two of the four paralogs in *H. marismortui*. In 10 out of 17 species the DUF439 gene is adjacent to CheY.

The only archaeal *che *gene regions without a DUF439 homolog are the *che2 *regions of the *Methanosarcina *species. In *Methanosarcina barkeri *this is the only *che *region, as this species does not contain the part of the genome where the *che1 *region in *M. mazei *and *M. acetivorans *is located [59-61]. The *che *gene region of *M. barkeri *is peculiar in that it has lost *cheC*, which is present in all other archaeal *che *regions.

**Figure 6:**
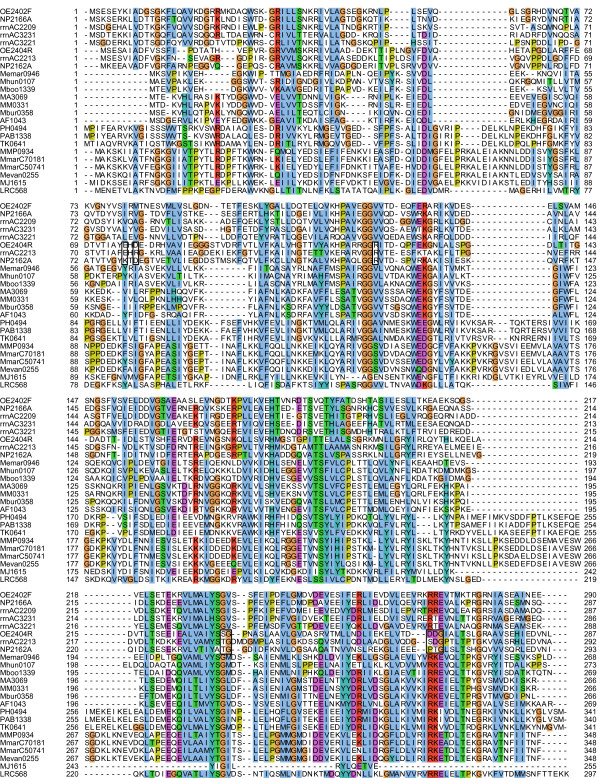
**Multiple alignment of the members of the protein family DUF439**. The species are: OE *Halobacterium salinarum *R1, NP *Natronomonas pharaonis*, rrn *Haloarcula marismortui*, Memar *Methanoculleus marisnigri*, Mhun *Methanospirillum hungatei*, Mboo *Candidatus Methanoregula boonei*, MA *Methanosarcina acetivorans*, MM *Methanosarcina mazei*, Mbur *Methanococcoides burtonii*, AF *Archaeoglobus fulgidus*, PH *Pyrococcus horikoshii*, PAB *Pyrococcus abyssi*, TK *Thermococcus kodakaraensis*, MMP *Methanococcus maripaludis *S2, MmarC7 *Methanococcus maripaludis *C7, MmarC5 *Methanococcus maripaludis *C5, Mevan *Methanococcus vannielii*, MJ *Methanococcus jannaschii*, LRC *uncultured methanogenic archaeon RC-I*. Colors are according to the ClustalX coloring scheme. The boxes point to peculiarities of the second DUF439 protein of the haloarchaea.

A multiple alignment of all members of the family DUF439 revealed only few conserved residues and several weakly conserved regions (Figure 6). No conserved motif could be detected that could provide a clue to the function of these proteins. It is noteworthy that in comparison to the other species the protein from *Methanocaldococcus jannaschii *(which lacks Che proteins) is less conserved and truncated at the C-terminus.

**Figure 7:**
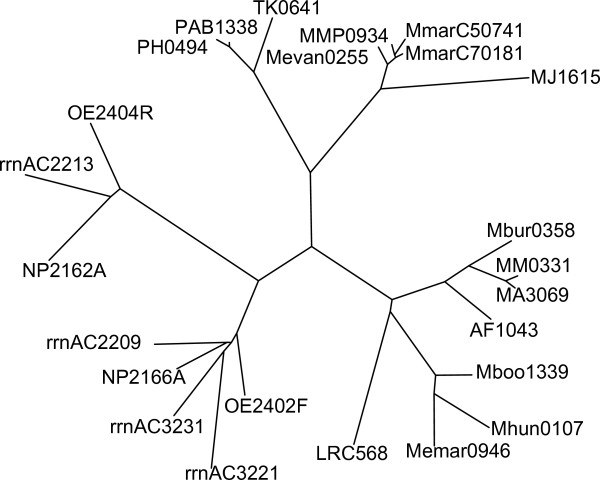
**Phylogenetic analysis of DUF439 proteins**. Unrooted phylogenetic tree by neighbor-joining, calculated from the multiple alignment shown in Figure 6. Species can be derived from the prefix of the protein identifier as explained in the legend of Figure 6.

Two or more copies of DUF439 proteins were only found in the motile haloarchaea *H. salinarum*, *N. pharaonis*, and *H. marismortui*. All three species contain a second homolog in or adjacent to the *che *gene region (OE2404R in *H. salinarum*). These second homologs lack several residues conserved in all other proteins of the family DUF439 (see boxes in Figure 6), and probably fulfill a different function than the main group of DUF439 proteins. This is consistent with the phenotypic results obtained for the deletions: the deletion of OE2404R resulted, other than the deletion of OE2402F, only in a weak phenotype. Phylogenetic analysis (Figure 7) revealed that the second homologs in the *che *gene region of the haloarchaea (OE2404R, NP2162A, rrnAC2213) form a separate branch in the phylogenetic tree, indicating that they probably arose by a gene duplication prior to the divergence of the haloarchaea. *H. marismortui *contains two additional DUF439 homologs located apart from the *che *gene region. These two paralogs resemble more the main group of DUF439 proteins than the second homolog of the haloarchaea, as can be seen in the multiple alignment and the phylogenetic tree. If they also fulfill a function in taxis signaling, it remains elusive.

## Discussion

### OE2401F, OE2402F, and OE2404R build a link between the Che system and the flagellar apparatus

Protein-protein interaction analysis in *H. salinarum *identified the proteins OE2401F, OE2402F, and OE2404R as interaction partners of the chemotaxis proteins CheY, CheD, and CheC2, as well as the flagella accessory proteins FlaCE and FlaD. The function of the flagellar accessory proteins is not known but their critical role in flagellation has been demonstrated [41,43,62,63]. The FlaE part of FlaCE is homologous to FlaD, both proteins contain a FlaD/E domain [58]. In *Methanococcus maripaludis*, the deletion of *flaC *resulted in non-motile and non-flagellated cells [44]. Deletion of *flaCE *and *flaD *in *H. salinarum *resulted in cells with a reduced number of flagella, which are hardly (Δ*flaD*) or not (Δ*flaCE*) motile [55]. Thus, Δ*flaCE *cells (and perhaps also Δ*flaD *cells) most likely have defects both in flagellar assembly and in flagellar function. These findings were interpreted as indicating that FlaC, FlaCE, and FlaD either function in flagellar secretion and assembly or that they are part of the flagellar motor or related structures. As mentioned in [44], in crenarchaeal genomes the genes *flaC-E *are generally absent (see also [42] and Additional file 6) although several crenarchaeal species are known to possess functional flagella, making a function assignment for these proteins even more difficult. However, in no crenarchaeal genome have *che *genes been detected (see Additional file 6), and we are not aware of any study reporting that a crenarchaeote reverses the flagellar rotational direction. Temperature-sensitive motility is described for *Sulfolobus acidocaldarius *[64], but this organism achieves reorientation by briefly halting its flagella and not by reversals [64,65]. This fact, and the connection to the response regulator CheY via the proteins identified in this study suggest that FlaC-E might be components of the flagellar motor or associated structures and might be involved in flagellar motor switching.

In bacteria, the link between the Che system and the flagellar motor is built by the interaction of CheY-P with the flagellar motor switch protein FliM. The archaeal flagellar motor is built from different components and driven by ATP instead of proton influx [37], but its overall function is the same.

Accordingly, it can be speculated that OE2401F, OE2402F, and OE2404R are either part of the archaeal flagellar motor switch, or they are adapters which fit the bacterial-like Che system to the yet unidentified archaeal switch.

OE2401F, OE2402F, and OE2404R also interact with CheD, and OE2402F and OE2404R with CheC2. In *B. subtilis*, CheC is a CheY-P phosphatase localized at the signaling complex [25]. CheD deamidates glutamine residues of the receptors and is necessary for receptor activation of CheA [66]. Together, these proteins build a feedback loop from the output of the system to the receptors [22]. Besides CheC, *B. subtilis *expresses with FliY a second CheY-P phosphatase, which is localized at the flagellar motor switch [25]. Generally, phosphatase localization turns out to be a conserved and important principle in bacterial chemotaxis systems [67]. In *H. salinarum*, receptor deamidase activity was demonstrated for the CheB protein, but not detected for CheD [68] and the cellular role of CheD and the three CheCs is unknown. However, provided that OE2402F and OE2404R are part of or related to the flagellar motor switch, the interaction with CheC2 might reflect CheY-P phosphatase localization similar to *B. subtilis*. CheC2 would then fulfill the role of FliY, and one or both of the other CheCs the role of *B. subtilis *CheC. Altogether, the protein interaction data are not sufficient to functionally characterize OE2401F, OE2402F, and OE2404R, but they provide strong evidence that these proteins act between the Che system and the archaeal flagellar apparatus.

### Without OE2401F and OE2402F the Che system and the flagellum are decoupled

The phenotypic characteristics of the deletion strains (see Table 3 for an overview) demonstrated that OE2401F and OE2402F are essential for the ability to control the direction of flagellar rotation, whereas the role of OE2404R remained unclear. The Δ4 strains were not distinguishable from wildtype strains in the phototaxis measurement and with respect to the flagellar rotational bias, but produced significantly smaller swarm rings. Hence, while it can be said that OE2404R is involved in taxis signal transduction in *H. salinarum*, it either fulfills a non-essential function or it can be replaced by its homolog, OE2402F, with only minor constraints.

**Table 3:** Phenotype of the deletion strains

	Δ**1**	Δ**2**	Δ**4**	Δ**2–4**
Motility	+	+	+	+
Chemotaxis	-	-	(+)	-
Phototaxis	-	-	+	-
CCW rotation	-	-	+	-

Cells of the strains Δ1, Δ2, Δ2–4 displayed very weak or no spontaneous switching, they did not respond to repellent light stimulation, and were unable to form swarm rings. They rotated their flagella almost exclusively clockwise. None of the strains exhibited defects in flagellar motility. Hence they behaved exactly like CheY and CheA deletion strains [35,54].

The data suggest that without OE2401F or OE2402F the Che system and the flagellum are decoupled. This could occur if either the Che system cannot generate its output, CheY-P, or if CheY-P is present but not effective. The first of these two possibilities seems less likely because the PPI data suggest a role for OE2401F and OE2402F between CheY and the flagellum, and not upstream of CheY. Additionally, the homology of the Che system to bacteria argues against the first hypothesis: Our current understanding is that the Che system of *H. salinarum*, with the ten known Che proteins, is complete up to CheY-P. Only for the part downstream of CheY-P have no homologs to bacterial proteins been found.

A further possibility to explain the behavior of Δ1, Δ2, Δ2–4 is an influence of the deleted proteins on the switch factor fumarate, which might act independently of the Che system. A defect in fumarate signaling can cause a phenotype similar to the one observed for Δ1, Δ2, Δ2–4 [46]. However, the detected protein interactions with CheY provide strong evidence that the proteins examined here play a role in the action of CheY and not exclusively in fumarate switching.

### OE2401F and OE2402F act cooperatively

Bioinformatics analysis did not reveal much knowledge for OE2401F. The PPI data suggest that OE2401F and OE2402F act cooperatively to perform their function. This idea is also supported by the genomic location of OE2401F and its homologs in the haloarchaeal *che *gene regions, where it is always adjacent to a DUF439 protein. However, in the chemotaxis gene regions of other archaeal species no homologs of OE2401F were found. Hence it remains to be investigated if these proteins are restricted to haloarchaea, or if similar proteins, coded elsewhere in the genome, play a role in taxis signaling also in other archaeal species.

### OE2402F and OE2404R belong to a family of archaea-specific Che proteins

The proteins OE2402F and OE2404R belong to the protein family DUF439 [58]. Proteins of this family were found to be an integral part of archaeal chemotaxis gene regions; they were not detected in other genomic contexts. The DUF439 gene is adjacent to *cheY *in 10 of 17 *che *gene regions, which supports the interaction found between these proteins [69].

The only archaeal chemotaxis gene regions without a DUF439 protein are the *che2 *regions of the three *Methanosarcina *species. Although these species are described as non-motile [70], they probably have the capability to swim by flagella since their genomes contain flagellins and a complete set of *fla *genes (see [42], Additional file 6). Whether the *Methanosarcina che2 *region plays a role in controlling flagellar motility and, if so, how this is done without DUF439 protein, remains to be elucidated.

Among the archaea with published genome sequences, *Methanocaldococcus jannaschii *is the only species which codes for a DUF439, but not for Che proteins. However, the protein from *Methanocaldococcus jannaschii *is less conserved and truncated at the C-terminus while this is well conserved in all other species. Hence it is likely that this protein is either non-functional or fulfills a different function.

The presence of a DUF439 protein in (almost) all archaeal *che *gene regions and the restriction to this genomic context indicate that these proteins constitute a hitherto unrecognized family of archaeal chemotaxis proteins.

## Conclusion

Overall, the PPI data and the observed deletion phenotypes strongly support a model where, in *H. salinarum*, CheY-P cannot trigger flagellar motor switching without OE2401F and OE2402F. Bioinformatics analysis has demonstrated that proteins of the DUF439 family are not only essential for chemo- and phototaxis in *H. salinarum*, but comprise a family of general archaeal chemotaxis proteins. The Che proteins in archaea were identified by homology to their bacterial counterparts [4-6], so the absence of DUF439 in bacteria might explain why these proteins were not recognized earlier. Since the archaeal chemotaxis proteins identified in this study connect the chemotaxis system to the archaeal flagellum, we propose the name CheF for this protein family. Future experiments will be necessary to determine the exact role of CheF in archaeal flagellar motor switching.

## Methods

### Strains and growth conditions

*H. salinarum *strains R1 (DSM 671) and S9 [71] were grown aerobically either in complex medium or in synthetic medium as described previously [72,73]. Transformed cells were grown with 10 μg ml^-1 ^mevinolin or 0.15 μg ml^-1 ^novobiocin. Transformation of *H. salinarum *was performed essentially as described by [74]. *E. coli *strain DH5*α *and transformants were grown in LB medium at 37°C and supplemented with ampicillin (100 μg ml^-1^), kanamycin (25 μg ml^-1^), or chloramphenicol (50 μg ml^-1^), if necessary.

### Protein-protein interaction analysis

Interactions between halobacterial proteins were determined by affinity purification of halobacterial protein complexes using bait proteins fused to a cellulose-binding domain. Components of the complex were identified by mass spectrometry. Additional file 1 provides a detailed description of this method.

### Construction of in frame deletion mutations

In-frame deletion plasmids were constructed using the vectors pMKK100 [50] and pMS3 (unpublished). All PCR reactions were done with Phusion Polymerase according to supplier's instructions and genomic DNA of *H. salinarum *strain R1 as template. 500 bp of sequence upstream (us) and downstream (ds) of the targeted gene were amplified by PCR using the primers listed in Additional file 7. The corresponding PCR products were used as templates in a second PCR using the external primers (us_fo and ds_re), resulting in a fusion product of us and ds sequence. The fusion products were ligated into both pMS3 and pMKK100, and the resulting deletion plasmids verified by DNA sequencing of the insert.

Deletion mutants were generated by transformation of the deletion plasmids into the wild type strains R1 and S9 and subsequent cultivation without selection pressure as described in [50]. Briefly, after transformation and plating on X-gal and antibiotic containing plates two blue clones were picked and grown in complex medium without antibiotics. After three passages of the culture, roughly 600 cells were plated on X-gal containing plates without antibiotics. Red colonies (red color indicates that these cells have lost the integrated plasmid) were inoculated into complex medium and screened for the loss of the target gene by PCR using the primers spanning the flanking regions.

### Southern blot analysis

Deletions were verified by Southern blot analysis. Genomic DNA of wild type and deletion strains was isolated and digested with BglI. DIG-labeled DNA probes were generated via PCR amplification of the upstream or the gene sequence from genomic DNA in the presence of DIG-11-dUTP (Roche). The digested DNA was subjected to 1% agarose gel electrophoresis, blotted to Hybond-N nylon membrane (Amersham Biosciences) and then UV-crosslinked. The use of blocking reagent, hybridization procedure and chemiluminescent detection with CSPD chemiluminescent substrate (Roche) was according to standard protocols.

### Complementation of deletions

Deleted genes were reintroduced into all deletion strains *in cis*. Complementation plasmids for each deletion were constructed by PCR amplification of the deleted gene(s) together with the flanking regions from *H. salinarum *R1 genomic DNA using the external primers (us_fo, ds_re) used for deletion plasmid construction. Inserts were digested with the respective restriction enzymes and cloned into pMS3, and the resulting plasmids were verified by sequencing of the insert. Each deletion strain was transformed with the corresponding complementation plasmid, and a double crossover triggered as described above. Red colonies were inoculated into complex medium and screened for reintroduction of the target gene by PCR using the primers spanning the flanking regions.

### Quantitative Realtime RT-PCR

Total RNA from 5 ml late log-phase cultures was isolated using the peqGOLD RNAPure™ system according to manufacturer's instructions. 3 μg total RNA were reverse transcribed with 50 pmol random hexamer primer (Applied Biosystems, Darmstadt, Germany) using Superscript III (Invitrogen, Karlsruhe, Germany). The quantitative PCR reactions were done in a GeneAmp 5700 Sequence Detection System (Applied Biosystems) using the SYBR Green PCR Master Mix Kit (Applied Biosystems). The final reaction volume was 25 μl with 0.5 μl of the reverse transcription reaction as template. Primers (see Additional file 7) were applied in a final concentration of 0.5 μM. Controls without template and control reactions amplifying a non-coding DNA region (the *bop *promoter) were included. The PCR consisted of 10 min initial denaturation at 95°C and 40 cycles of 15 sec 95°C and 1 min 60°C. Uniformity of the product was assured by measuring the melting curve of the product. Transcript level differences were calculated by the ΔΔ*C*_*t *_method using the constitutively expressed *fdx *gene (OE4217R) as internal standard. For all calculations the mean-*C*_*t *_of 2 replicate reactions was used. Results were accepted if the *C*_*t *_of both replicates differed by less than 0.5, and if the difference to the lowest *C*_*t *_of the controls was at least 5.

### Swarm plates

Semi-solid agar plates were prepared from complex medium with 0.25% agar. Wild type and deletion cultures were grown to an OD_600 _of 0.6 – 0.8. Fresh medium was inoculated with equal amount of cells from the starter cultures and culturing repeated twice to achieve equal cell densities in the final cultures. 10 μl of culture with an OD_600 _of 0.6 – 0.8 were injected with a pipette tip into the soft agar. The plates were incubated for 3 days at 37°C in the dark.

### Computerized cell tracking (Motion analysis)

Reversal frequencies of unstimulated cells and after application of a photophobic stimulus were measured with a computerized cell tracking system [52]. The system consists of a phase-contrast microscope (BX51, Olympus) equipped with a CCD camera (COHU, USA) which allows stimulus-free observation of the cells using infrared light. To measure the responses to light stimuli, the light from two computer-controlled light sources (MT20-SPA, Olympus) was applied to the cells. Cells were grown in 35 ml complex medium to an OD_600 _of 0.6 – 0.9. Cells were diluted with complex medium and arginine to an OD_600 _of 0.32 and a final arginine concentration of 0.1% (w/v). Diluted cells were incubated in the dark at RT for at least 20 min. For measurement, 5 μl cell suspension were pipetted on a slide and sealed under a cover slip with a molten 2:1 (w/w) mixture of paraffin wax and vaseline. Before starting the measurements, the specimen was incubated for 5 min on the heated stage (25°C). An experiment consisted of 20 single measurements, each recording 5 s of cell movement. From this a 4 s interval was analyzed for cell reversal.

For measuring the blue light response, a blue light pulse (480 ± 50 nm excitation filter, 0.5 s duration, 5% intensity) was applied through the objective at the beginning of the tracking interval. After each measurement the position on the slide was changed to avoid repeated stimulation of the same cells. For measurement of the response to an orange light step-down, the cells were initially adapted for 5 min to orange light (580 ± 50 nm excitation filter, applied through the condenser). At the beginning of the tracking interval, the orange light was switched off for 4 s. Prior to each subsequent measurement, the cells were adapted again for 45 s.

Reversals are detected by an algorithm based on a Kalman filter [52]. Briefly, for each time point, a prediction of the cell position for some time span in the future is made based on the last measurements. The prediction is compared with the actual position after the time span has elapsed. Reversals are detected by this comparison (see also [31]) with a false positive and false negative rate of 2 and 2.5% [52], respectively. The 95% confidence intervals were calculated assuming a binomial distribution according to Lorenz [75].

By measuring known straight-swimming mutants (cheY**, [35]), the false positive detection of reversal events (tracking error) was determined to be maximally 2.5–5% in a 4 s observation interval [52].

### Dark-field microscopy

To visualize the flagellar bundle, cells were investigated on a dark-field microscope (Olympus BX50, equipped with an USH-120D mercury lamp and U-DCW cardioid immersion dark-field condenser). Cell culture and preparation of microscopic specimens was done as described above. Cells were diluted to an OD_600 _of 0.1 with complex medium and arginine added to a final concentration of 0.1%. 50 μl immersion oil (n_*e *_= 1.5180, Leitz, Wetzlar, Germany) were pipetted on the condenser, the slide put onto the stage, and the condenser adjusted to maximal height. The cells were focused and the condenser gradually lowered again (with permanent refocusing), until the flagella became visible. Each specimen was used for one hour at the most. The flagellar rotational bias was determined by counting the cells swimming with the flagellum in front of the cell body (CCW) and cells swimming with the flagellum behind the cell body (CW). Bipolarly flagellated cells were excluded from the analysis. Cells which changed their swimming direction during observation were counted with the first swimming direction.

### Bioinformatic analysis

The multiple alignment of the DUF439 proteins was calculated using ClustalX [76,77] using standard parameters. For phylogenetic analysis, a neighbor-joining tree was calculated from the multiple alignment applying the Phylip package [78]. Again, standard parameters were used.

## Abbreviations

che: chemotaxis; CCW: counterclockwise; CW: clockwise; DUF: domain of unknown function; ds: downstream; fla: flagella accessory; Htr: halobacterial transducer; MCP: methyl-accepting chemotaxis protein; PPI: protein-protein interaction; qRT-PCR: quantitative Realtime Reverse Transcription PCR; SILAC: stable isotope labeling with amino acids in cell culture; us: upstream

## Authors' contributions

MS and DO conceived and designed the experiments. AM, JM, and MS performed the bait-fishing experiments. BS and FS performed the mass spectrometric measurements, MS and AM analyzed the MS data. AM created the deletion mutants, JM and AM the complementations. AM performed the swarm-plate assays, the cell-tracking experiments, and the dark-field microscopy with help from WS and SS. SS analyzed the cell-tracking data. AM performed the qRT-PCR experiments. MS performed the computational analysis. MS produced the figures and wrote the manuscript. SS, WS, FS, and DO revised the manuscript. All authors read and approved the final manuscript.

## Supplementary Material

Additional File 1 **Protein-protein interaction analysis.** This file provides additional information about the protein-protein interaction analysis. There are a figure and a table (Figure S1 and Table S1) detailing the results presented in Figure 2. Additionally, a figure illustrating the applied methods (Figure S2) and a detailed description of the methods are included. Click here for file

Additional File 2 **Confirmation of deletion strains by Southern blot analysis.** Each deletion strain was probed with DIG-labeled 500 bp upstream sequence of the target gene(s) (us probe) and DIG-labeled target sequence (gene probe). Deletion strains are labeled according to their host strain (R1 or S9) followed by a Δ and the last digit of the identifier(s) of the deleted gene(s). 1 and 2 indicate the clones of the respective deletion that showed the expected bands and were used for further analysis, wt indicates the corresponding wild type. The upstream probe for OE2401F revealed an additional band, probably due to unspecific binding. This band, however, did not affect the significance of the blot. Click here for file

Additional File 3 **Swarming ability of the deletion strains.** Swarm plates for the deletion strains in R1 and S9 background are shown. On each plate, the deletion strain (bottom) is compared to the respective wildtype strain (top). For each deletion in both host strains, two clones were tested (C1 and C2). Each clone was examined on two plates. Click here for file

Additional File 4 **Results of computer-based cell-tracking experiments.** This table contains the detailed results from the computer-based cell-tracking experiments. Click here for file

Additional File 5 **Phenotype of complementations. A **Swarm plate assay. On each plate the complementation strain (bottom) is compared to the respective wildtype strain (top). **B **Computer-based cell tracking for the complementations of each single deletion. The percent reversal in a 4 second interval was determined either without stimulation (spontaneous, gray bar) or after a blue light pulse (blue bar). Error bars represent the 95% confidence interval. Click here for file

Additional File 6 **Occurrence of *che *and *fla *genes in archaeal genomes.** An exhaustive search for *che *and *fla *genes in archaeal genomes is presented and the detected orthologs listed as table (Table S2). Additionally, the method used for ortholog identification is described. Click here for file

Additional File 7 **Primers used in this study.** This table lists the oligonucleotides used in the present study. Click here for file
