# Supplementary material for: Identification of Archaea-specific chemotaxis proteins which interact with the flagellar apparatus
Source: BMC Microbiol. 2009 Mar 16;9:56. doi: 10.1186/1471-2180-9-56 (PMC2666748; doi:10.1186/1471-2180-9-56)
Supplement: Additional File 1 — Protein-protein interaction analysis. This file provides additional information about the protein-protein interaction analysis. There are a figure and a table (Figure S1 and Table S1) detailing the results presented in Figure 2. Additionally, a figure illustrating the applied methods (Figure S2) and a detailed description of the methods are included. [file 1471-2180-9-56-S1.pdf]

Supplementary Material - Protein-protein interaction analysis

Interactions between halobacterial proteins were identified by affinity purification combined with mass spectrometry (AP-MS). As affinity tag a cellulose-binding domain from *Clostridium thermocellum* was used [1], which binds cellulose even in the presence of multi-molar salt concentrations, so the whole purification can be performed under close to physiological conditions [2, 3]. Labeling with stable isotopes (SILAC, [4]) was applied to discriminate specific interaction partners from unspecific contaminants. Affinity purification experiments with the ten halobacterial Che proteins and, till now, seven associated proteins, have been done (Schlesner *et al.*, in preparation). The results of the experiments relevant to this study are summarized in Figure 2. Details are given in Figure S1 and Table S1.

Neither the proteins OE2401F, OE2402F and OE2404R nor FlaCE and FlaD were found as interaction partners in any of the other affinity purification experiments performed so far (unpublished data).

Table S1: **Protein identification in PPI analysis.**

Protein	Bait	Method	Prob	Id pep	Ass Score	Quan pep
FlaCE	OE2402F	indirect	1.00	2	20.04	1
FlaCE	OE2402F	direct	1.00	2	11.76	1
FlaCE	OE2404R	indirect	1.00	3	9.57	1
FlaD	OE2404R	indirect	1.00	3	12.85	2
OE2402F	OE2401F	direct	1.00	7	12.37	3
OE2402F	CheD	direct	1.00	2	10.58	1
OE2402F	CheY	direct	1.00	5	10.84	2
OE2402F	CheC2	indirect	1.00	4	22.42	1
OE2404R	CheD	direct	1.00	3	14.62	2
OE2404R	CheY	indirect	1.00	2	16.26	1
OE2404R	CheC2	indirect	1.00	4	25.87	1
CheD	OE2401F	direct	1.00	2	37.69	1
CheD	CheC2	indirect	1.00	2	10.28	1
CheA	CheY	indirect	1.00	9	60.10	6

Proteins relevant to this study identified with the indicated baits in direct or indirect bait fishing experiments are listed. The columns Prob, Id pep, Ass score, and Quan pep give the ProteinProphet [5] probability, number of identified peptides, association score, and number of quantified peptides.

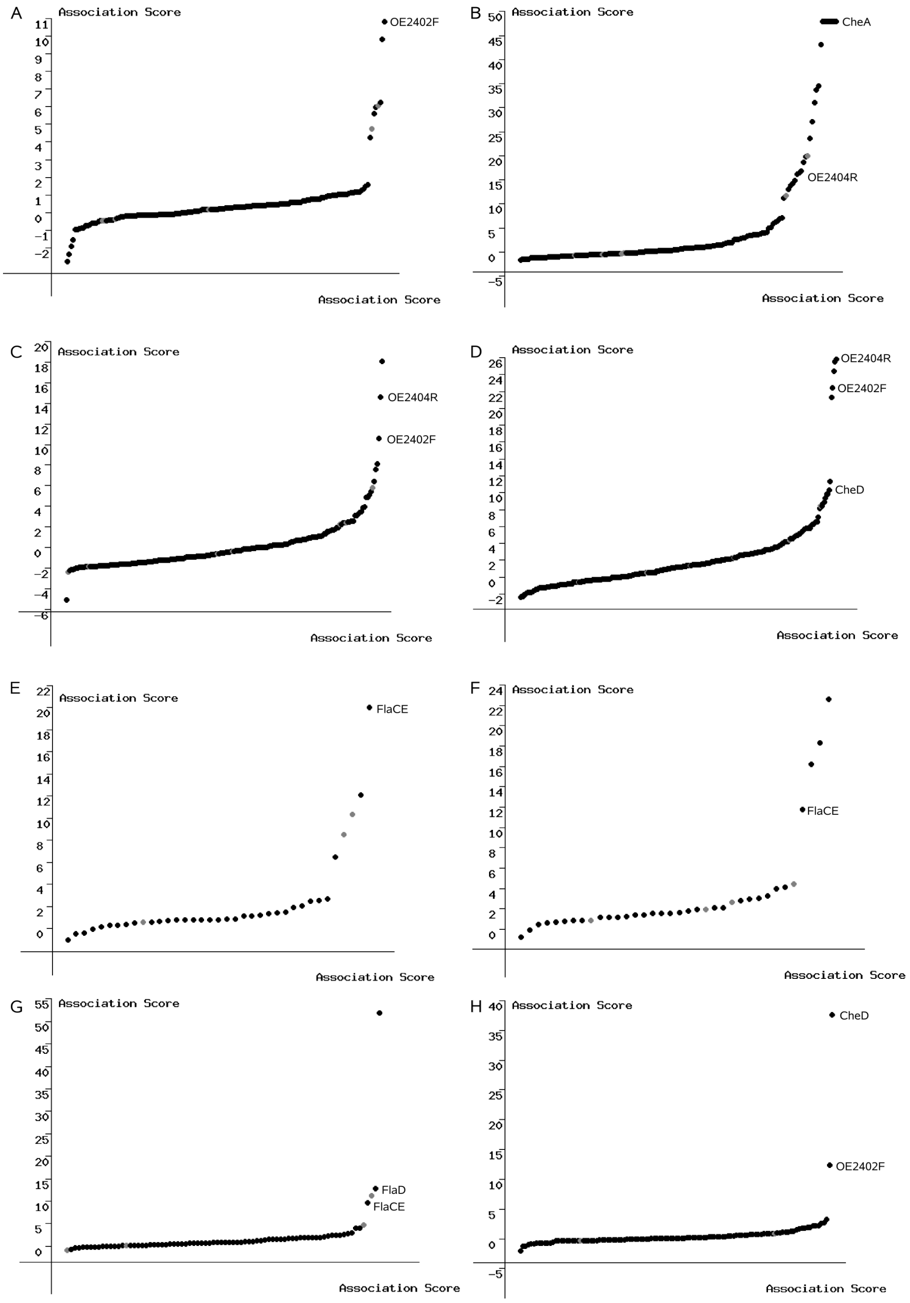

Figure S1: **Protein-protein interaction identification.** Distribution of the association scores of the proteins identified in the affinity purification experiments. Identified Che and Fla proteins, and the proteins OE2401F, OE2402F, and OE2404R are indicated. The baits (method) were **A** CheY (direct), **B** CheY (indirect), **C** CheD (direct), **D** CheC2 (indirect), **E** OE2402F (indirect), **F** OE2402F (direct), **G** OE2404F (indirect), **H** OE2401F (direct). Grey points indicate proteins considered as contaminants (binders of the CBD, the empty cellulose column, or promiscuous binders).

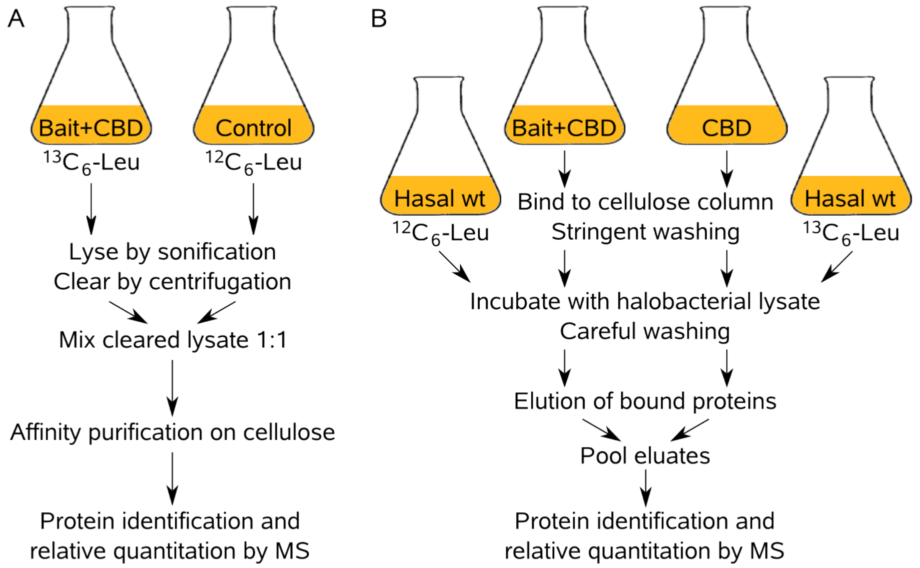

Figure S2: **Schematic of purification procedures applying SILAC. A** Direct bait fishing. The bait expression strain (transformed with the bait's pMS4 construct) and the control strain (transformed with the bait's pMS6 construct) are grown in synthetic medium containing ^{13}C-leucine or ^{12}C-leucine, respectively. The lysate from both strains is mixed and purification done on one cellulose column. **B** Indirect bait fishing. The bait expression strain and the control strain (expressing the plain CBD) are grown in complex medium. Bait and CBD are bound to separate cellulose columns and stringently washed in order to remove all proteins except bait or CBD. The columns are incubated with halobacterial lysate from cells grown in synthetic medium containing ^{12}C-leucine (bait) or ^{13}C-leucine (pMS4), respectively. After elution, the eluates are pooled.

Methods

Two variations of the affinity purification method, called direct and indirect bait fishing, were developed (Figure S2). Since both methods were found to have strengths and weaknesses in certain cases, they were applied both to every analyzed protein.

Generation of *H. salinarum* expression strains

Plasmids for expression and purification of CBD-tagged bait proteins were generated using the Gateway recombinational cloning system (Invitrogen). Bait proteins were PCR amplified using the primers listed in Additional file 6 with Phusion polymerase (Finnzymes) according to supplier's recommendations. The purified PCR products were cloned into the pENTR/D-TOPO vector (Invitrogen) according to manufacturer's instructions, and transformed into *E. coli* DH5α competent cells. Kanamycin-resistant (kanR) colonies were screened by colony PCR using the primers M13F and M13R to verify the correct insert size, and positive clones sequence-verified using the same primers. Using Gateway LR clonase reactions (Invitrogen), pENTR clones were recombined with the destination vectors pMS4 (*H. salinarum* CBD fusion expression vector, unpublished) and pMS6 (*H. salinarum* bait control expression vector, unpublished) to create expression plasmids for either the bait protein or the corresponding control.

Expression plasmids were transformed into *H. salinarum* R1. The plasmids do not contain an origin of replication for *H. salinarum* but are integrated into the genome at the locus of the bait protein by homologous recombination. Expression of the tagged bait protein was verified by affinity purification. Control strains transformed with pMS6 were checked by western blot with an anti-penta-his HRP conjugate (QIAGEN) using the XCell II™ Blot Module (Invitrogen) and Lumi-Light Western Blot Substrate (Roche) according to manufacturer's instructions.

Affinity purification of CBD-tagged proteins

The bait expression strain was precultured in 35 ml Halomedium containing 0.15 µg /ml novobiocin at 37°C on a shaker (150 rpm) until an OD_{600} of 0.5-1.0 was reached. This preculture was used to inoculate 100 ml complex medium to an OD_{600} of 0.01. When the main culture had reached an OD_{600} of 0.6 to 1.0, cells were harvested by centrifugation (8000 rpm, 15 min, 15°C) and resuspended in 1-2 ml CFE buffer (3 M KCl, 1 M NaCl, 400 mM NH_4Cl, 40 mM $MgCl_2$, 10 mM Tris/HCl, pH 7.5) plus protease inhibitor (Complete Mini, EDTA-free, Roche) (CFE + PI). Cells were lysed by sonication on ice water (2 x 20 sec, Branson sonifier 250, 3 mm disruptor horn, output level 2, constant), and the

lysate cleared by centrifugation at 14000 rpm, 18°C for 20 min in a tabletop centrifuge.

A cellulose column was prepared by pipetting 300 µl cellulose suspension, 10 % (w/v) Avicell PH-101 (Fluka) in CFE, into a Mobicol empty spin column (MoBiTec). The column was centrifuged (300 x g, 1 min, RT), washed with 600 µl CFE to remove fines, and centrifuged again.

The cleared lysate was applied to the column in 600 µl portions and the cellulose resuspended by pipetting up and down. After 1 min incubation at room temperature, the column was centrifuged (300 x g, 1 min, RT) and the flow-through discarded. The cellulose was washed three times with 600 µl CFE + 0.5 % NP40 (Roche) and once with CFE. After each washing step the column was centrifuged (300 x g, 1 min, RT) and the flow-through discarded. An additional centrifugation (770 x g, 1 min, RT) was performed after the last washing step to reduce the amount of retained buffer. For elution, 600 µl ethylene glycol were applied to the column, the cellulose resuspended, and the column centrifuged. Eluted proteins were precipitated with TCA. For this, an equal volume of 20 % (w/v) TCA was added, the mixture incubated on ice for 30 minutes, then centrifuged at 14000 rpm, 4°C, and the pellet washed 2-3 times with ice-cold 50 % (w/v) acetone.

For SILAC-based direct bait-fishing experiments the above protocol was modified as follows:

The bait expression strain and the control strain were precultured in 35 ml Halomedium containing 0.15 µg /ml novobiocin at 37°C on a shaker (150 rpm) until an OD_{600} of 0.5-1.0 was reached. 500 µl of these first precultures were used to inoculate second precultures that were grown under identical conditions to an OD_{600} of 0.8-1.0. With both cultures 100 ml synthetic medium were inoculated to an OD_{600} of 0.01. The medium contained $^{13}C_6$-leucine for the bait expression strain and $^{12}C_6$-leucine for the control culture. To guarantee identical conditions for both the bait and the control culture, the inoculum for both cultures was brought to a total volume of 1.5 ml with complex medium.

The main cultures were incubated on a shaker (110 rpm) at 37°C in the dark until they had reached an OD_{600} of 0.8. In order to work with roughly the same number of cells from both cultures, differences in the OD of bait and control culture were compensated by reducing the volume used from the culture with higher density accordingly. Cells were harvested by centrifugation (8000 rpm, 15°C, 15 min) and pellets resuspended in 1 ml CFE + PI. Cell lysate and cellulose columns were prepared as described above. 300 µl lysate from each culture were applied to the column, the cellulose resuspended, and after 1 min incubation the column centrifuged (300 x g, 1 min, RT). This step was repeated twice, followed by washing, elution, and protein precipitation as described.

Indirect bait-fishing experiments were performed with the following modifications:

H. salinarum R1 was precultured twice in 35 ml Halomedium at 37°C on a shaker (110 rpm) until an

OD_{600} of 0.5-1.0 was reached. When the second preculture had reached an OD_{600} of 0.8-1.0 it was used to inoculate 2 cultures with 100 ml synthetic medium, one containing $^{13}C_6$-leucine, the other one containing $^{12}C_6$-leucine, to an OD_{600} of 0.01. The inoculum was brought to a total volume of 1.5 ml with complex medium. The cultures were incubated on a shaker (110 rpm) at 37°C in the dark until they had reached an OD_{600} of 0.8.

In parallel, the bait expression strain and the pMS4 control strain were precultured in 35 ml Halomedium containing 0.15 µg /ml novobiocin at 37°C on a shaker (150 rpm) until an OD_{600} of 0.5-1.0 was reached. A second preculture was grown as described before. When an OD_{600} of 0.8-1.0 was reached 200 ml complex medium were inoculated to an OD_{600} of 0.01 and incubated at 37°C on a shaker (110 rpm). The main cultures were harvested at an OD_{600} of around 1.0. Cells of all four cultures were pelleted and lysate prepared as described. Two cellulose columns were prepared as described above. 600 µl lysate from the bait expression culture and the pMS4 control culture were applied to one cellulose column each, the cellulose resuspended, and after 1 min incubation the columns were centrifuged (300 x g, 1 min, RT). This step was repeated, and the columns washed three times with CFE + 1% NP40 + 20% ethylene glycol and once with CFE.

Lysate from the *H. salinarum* R1 wt cells was applied to these columns in 600 µl portions (cells labeled $^{12}C_6$-Leucine for the bait column,$^{13}C_6$-Leucine for the control column) , the cellulose resuspended, and after 1 min incubation the column centrifuged (300 x g, 1 min, RT). Washing, elution and protein precipitation was done as described above.

Mass spectrometric identification of purified proteins

Precipitated proteins were separated on 4-12 % Bis Tris gels (NuPAGE, Invitrogen) and stained with Coomassie Brilliant Blue R250. For LC-MS/MS analysis, the whole lane was cut out of the gel and divided into 10-15 slices. Size of the slices was chosen according to the estimated amount of tryptic peptides derived from the respective part of the lane. Additionally, very thick bands were separated from weaker ones to prevent masking of low-abundance proteins. Slices were cut into pieces of circa 1 mm^3 and transfered to 0.5 ml reaction tubes. Digestion and elution were performed essentially as described by Shevchenko [6]. Peptides were desalted by reverse phase (RP) chromatography using self-packed Stage tips (STop And Go Extraction, [7]). Protein identification by nanoLC-MS/MS on a ESI Q-TOF Ultima mass spectrometer (Waters, Milford, MA) was done as described in [8] with minor modifications.

Briefly, peptides were chromatographically separated on a CapLC system (Waters) and the eluate

directly injected into a Q-TOF ultimate mass spectrometer (Waters). The dried peptides were dissolved in 20 µl 5 % formic acid, and 1-6 µl (depending on the amount of protein estimated by the intensity of the Coomassie stained gel) were loaded into the CapLC using an auto sampler. They were bound to the precolumn (self-packed, 100 µm x 25 mm ReproSil-Pur 200 C_{18}-AQ, 5 µm, Dr. Maisch GmbH, Ammerbuch-Entringen, Germany) with a flow of 2 µl/min and analyzed on the main column (self-packed, 75 µm x 150 mm ReproSil-Pur 200 C_{18}-AQ, 3 µm) with a flow of 200 nl/min. Bound peptides were eluted in an linear acetonitril gradient and injected into the mass spectrometer.

Mass spectrometric analysis was performed in the *positive ion mode* with a capillary voltage of 2.3 kV. The mass window was set to 300-2000 Da in MS mode and 50-2000 Da in MS/MS mode. Survey scans were acquired for 1.5 s. From each survey scan up to four peptides were chosen for fragmentation by CID (collision-induced dissociation); selection criteria were the signal intensity and the charge state (at least two-fold). CID was performed with a collision voltage between 16 and 40 kV and helium as collision gas.

Data analysis

Peak lists were extracted from the raw data with Mascot Distiller and submitted to an in-house Mascot server for search against a *Halobacterium salinarum* R1 protein sequence database. Carbamidomethylation of cysteine was set as a required modification and oxidation of methionine and acetylation of the protein's N-terminus as variable modification. Up to three missed cleavage sites were allowed. For SILAC experiments, $^{13}C_6$-Leucine were additionally set as variable modification. Mass tolerance was set to 1.5 Da for MS and 0.6 Da for MS/MS.

Protein ratios of SILAC experiments were determined with ASAPRatio [9] embedded in the Trans-Proteomic Pipeline [10]. ASAPRatioPeptideParser was used with the options "lL" (set leucine as labeled residue), "-C" (quantitate only the charge state where the CID was made), "B" (return a ratio even if the background is high), and "-F" (use fixed scan range for light and heavy peptide). All other tools were run with the default parameters. Protein ratios were checked manually on the basis of the extracted ion chromatograms and adjusted if necessary (e. g. background level or scan range). Only protein identifications with at least two different peptides, a ProteinProphet probability [5] of 0.95 or higher and a protein ratio (at least one of the identified peptides had to contain leucine, and the extracted ion chromatograms had to be utilizable by ASAPRatio) were accepted.

To accomplish a better presentability of the protein ratios a symmetrical measure, called Association Score, was introduced. Association Score was calculated from the SILAC ratio (bait isotopic form

divided by control isotopic form) as follows:

$$AssociationScore = \begin{cases} ASAPRatio - 1 & \text{if } ASAPRatio \geq 1 \\ 1 - \frac{1}{ASAPRatio} & \text{if } ASAPRatio < 1 \end{cases}$$

References

[1] Morag E, Lapidot A, Govorko D, Lamed R, Wilchek M, et al. (1995) Expression, purification, and characterization of the cellulose-binding domain of the scaffoldin subunit from the cellulosome of *Clostridium thermocellum.* Appl Environ Microbiol 61:1980–1986. URL http://www.pubmedcentral.nih.gov/articlerender.fcgi?tool=pubmed&pubmedid=7646033.

[2] Ortenberg R, Mevarech M (2000) Evidence for post-translational membrane insertion of the integral membrane protein bacterioopsin expressed in the heterologous halophilic archaeon *Haloferax volcanii.* J Biol Chem 275:22839–22846. doi:10.1074/jbc.M908916199. URL http://dx.doi.org/10.1074/jbc.M908916199.

[3] Irihimovitch V, Ring G, Elkayam T, Konrad Z, Eichler J (2003) Isolation of fusion proteins containing SecY and SecE, components of the protein translocation complex from the halophilic archaeon *Haloferax volcanii.* Extremophiles 7:71–77. doi:10.1007/s00792-002-0297-0. URL http://dx.doi.org/10.1007/s00792-002-0297-0.

[4] Ong SE, Blagoev B, Kratchmarova I, Kristensen DB, Steen H, et al. (2002) Stable isotope labeling by amino acids in cell culture, SILAC, as a simple and accurate approach to expression proteomics. Mol Cell Proteomics 1:376–386. URL http://www.ncbi.nlm.nih.gov/pubmed/12118079.

[5] Nesvizhskii AI, Keller A, Kolker E, Aebersold R (2003) A statistical model for identifying proteins by tandem mass spectrometry. Anal Chem 75:4646–4658.

[6] Shevchenko A, Wilm M, Vorm O, Mann M (1996) Mass spectrometric sequencing of proteins silver-stained polyacrylamide gels. Anal Chem 68:850–858. URL http://www.ncbi.nlm.nih.gov/pubmed/8779443.

[7] Rappsilber J, Ishihama Y, Mann M (2003) Stop and go extraction tips for matrix-assisted laser desorption/ionization, nanoelectrospray, and LC/MS sample pretreatment in proteomics. Anal Chem 75:663–670. URL http://www.ncbi.nlm.nih.gov/pubmed/12585499.

[8] Klein C, Garcia-Rizo C, Bisle B, Scheffer B, Zischka H, et al. (2005) The membrane proteome of *Halobacterium salinarum*. Proteomics 5:180–197. doi:10.1002/pmic.200400943. URL http://dx.doi.org/10.1002/pmic.200400943.

[9] Li XJ, Zhang H, Ranish JA, Aebersold R (2003) Automated statistical analysis of protein abundance ratios from data generated by stable-isotope dilution and tandem mass spectrometry. Anal Chem 75:6648–6657. doi:10.1021/ac034633i. URL http://dx.doi.org/10.1021/ac034633i.

[10] Keller A, Eng J, Zhang N, jun Li X, Aebersold R (2005) A uniform proteomics MS/MS analysis platform utilizing open XML file formats. Mol Syst Biol 1:2005.0017. doi:10.1038/msb4100024. URL http://dx.doi.org/10.1038/msb4100024.
