# Supplementary figures and images for: Identification of Archaea-specific chemotaxis proteins which interact with the flagellar apparatus
Source: BMC Microbiol. 2009 Mar 16;9:56. doi: 10.1186/1471-2180-9-56 (PMC2666748; doi:10.1186/1471-2180-9-56)

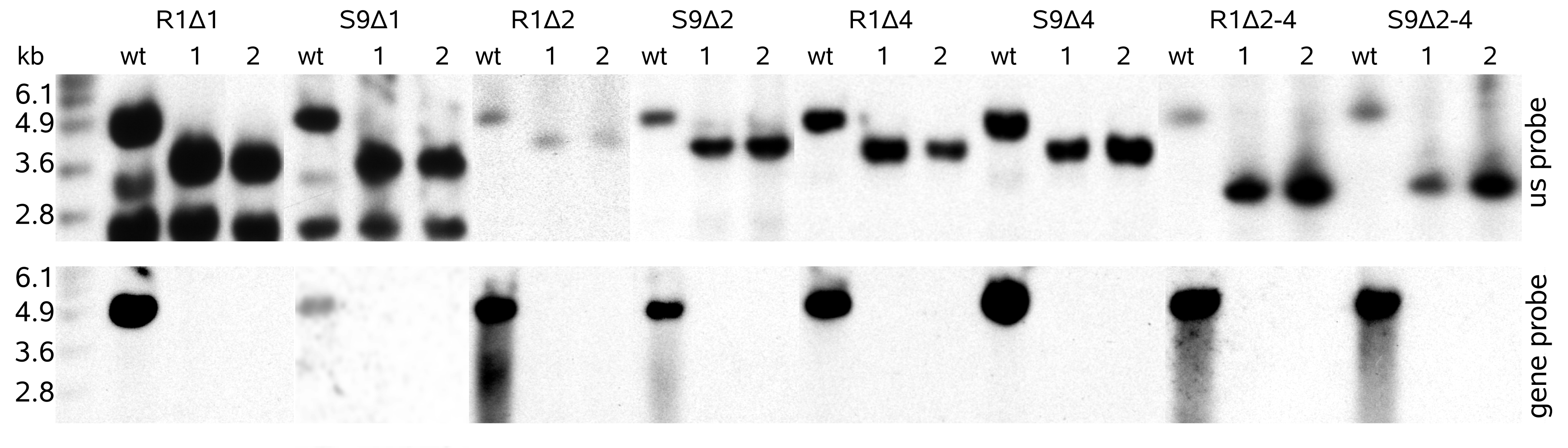

Supplement: Additional File 2 — Confirmation of deletion strains by Southern blot analysis. Each deletion strain was probed with DIG-labeled 500 bp upstream sequence of the target gene(s) (us probe) and DIG-labeled target sequence (gene probe). Deletion strains are labeled according to their host strain (R1 or S9) followed by a Δ and the last digit of the identifier(s) of the deleted gene(s). 1 and 2 indicate the clones of the respective deletion that showed the expected bands and were used for further analysis, wt indicates the corresponding wild type. The upstream probe for OE2401F revealed an additional band, probably due to unspecific binding. This band, however, did not affect the significance of the blot. [file 1471-2180-9-56-S2.png]

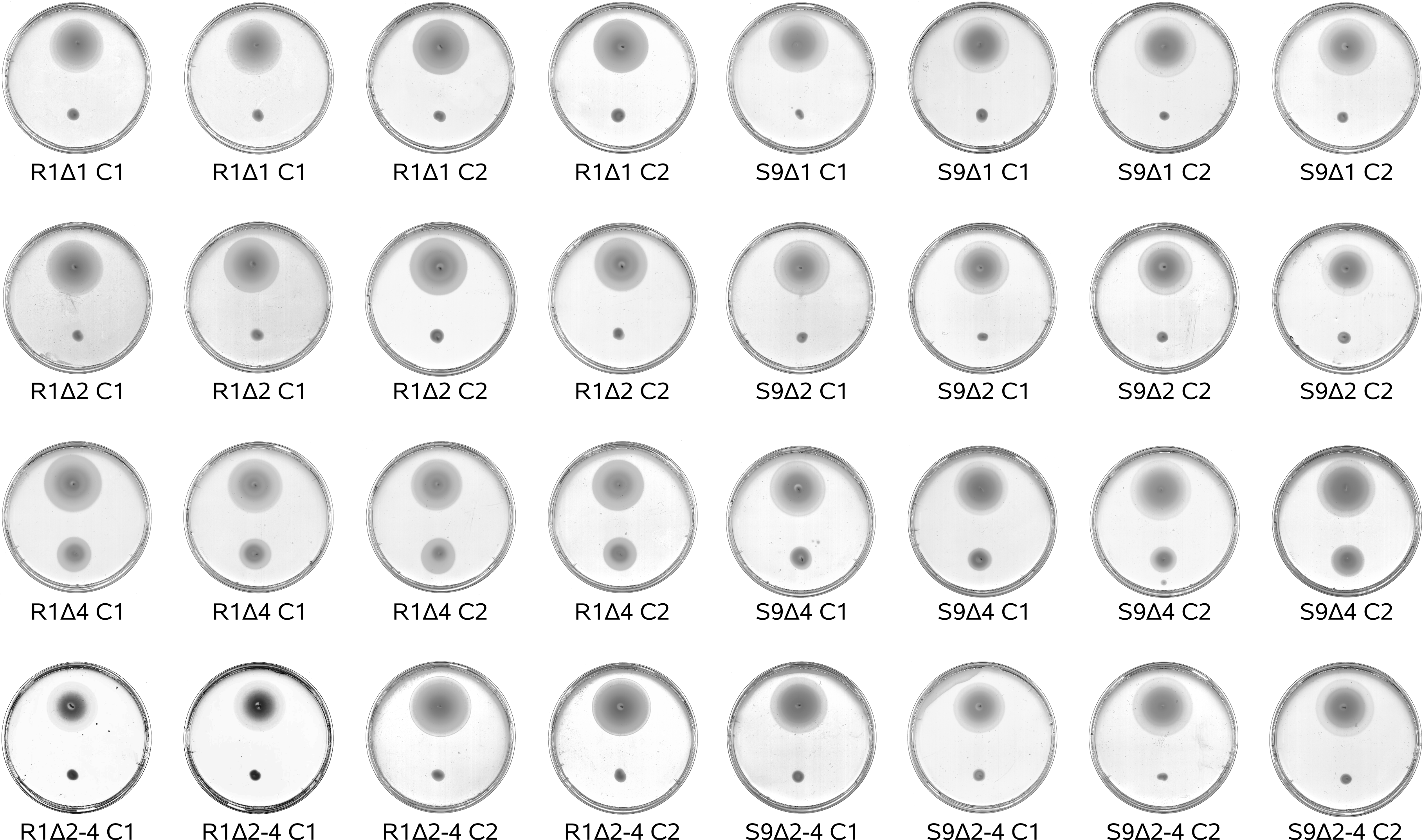

Supplement: Additional File 3 — Swarming ability of the deletion strains. Swarm plates for the deletion strains in R1 and S9 background are shown. On each plate, the deletion strain (bottom) is compared to the respective wildtype strain (top). For each deletion in both host strains, two clones were tested (C1 and C2). Each clone was examined on two plates. [file 1471-2180-9-56-S3.png]

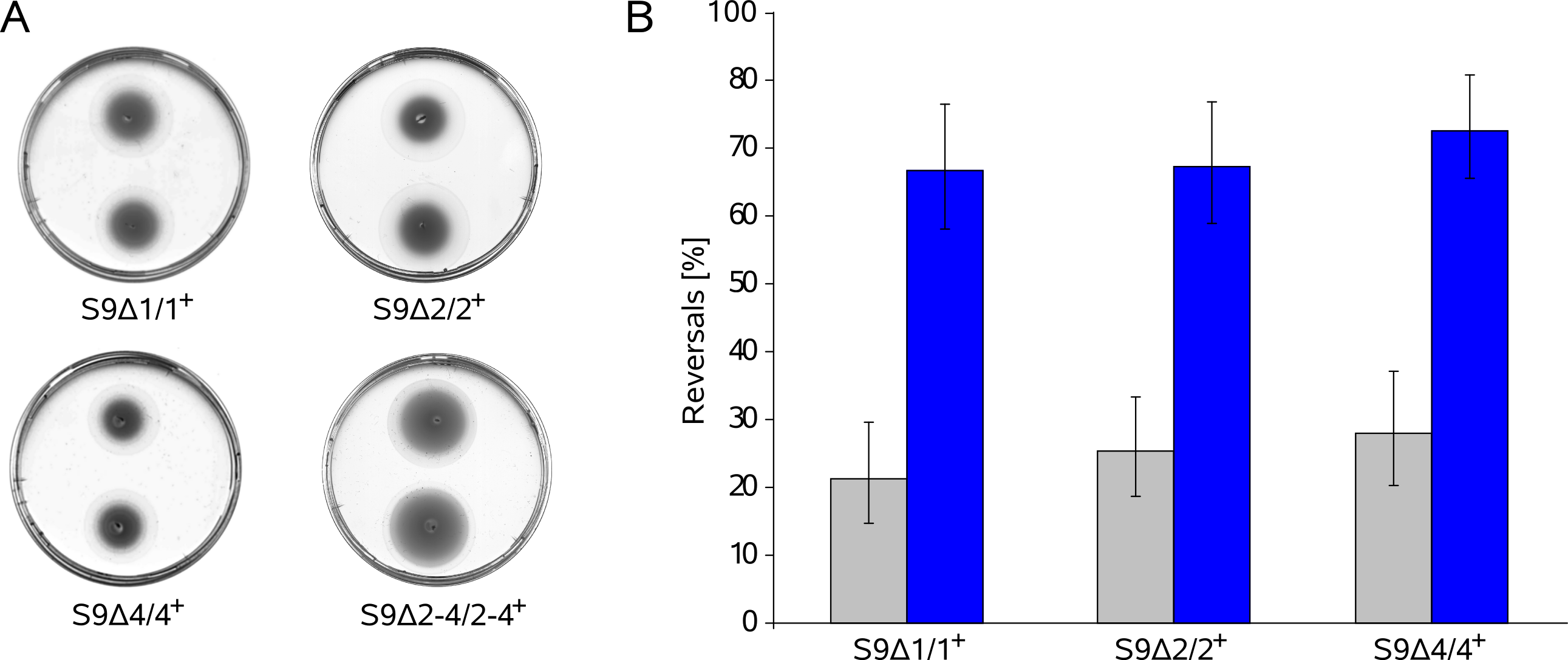

Supplement: Additional File 5 — Phenotype of complementations. A Swarm plate assay. On each plate the complementation strain (bottom) is compared to the respective wildtype strain (top). B Computer-based cell tracking for the complementations of each single deletion. The percent reversal in a 4 second interval was determined either without stimulation (spontaneous, gray bar) or after a blue light pulse (blue bar). Error bars represent the 95% confidence interval. [file 1471-2180-9-56-S5.png]
