# Supplementary material for: Identification of Archaea-specific chemotaxis proteins which interact with the flagellar apparatus
Source: BMC Microbiol. 2009 Mar 16;9:56. doi: 10.1186/1471-2180-9-56 (PMC2666748; doi:10.1186/1471-2180-9-56)
Supplement: Additional File 4 — Results of computer-based cell-tracking experiments. This table contains the detailed results from the computer-based cell-tracking experiments. [file 1471-2180-9-56-S4.pdf]

Results of computer-based cell-tracking experiments.

Clone		# Cells	# Reversals	% Reversals	P_l (%)	P_u (%)
	O	256	183	71.48	65.46	76.85
S9	B	162	111	68.52	60.69	75.45
	S	216	51	23.61	18.23	29.95
	O	127	100	78.74	70.41	85.29
R1	B	100	81	81.00	71.67	87.89
	S	74	7	9.46	4.21	19.09
	O	166	11	6.63	3.52	11.84
S9Δ1 C1	B	153	4	2.61	0.84	6.98
	S	201	3	1.49	0.39	4.65
	O	39	6	15.38	6.41	31.21
S9Δ1 C2	B	77	11	14.29	7.69	24.55
	S	201	20	9.95	6.33	15.16
	O	58	0	0.00	0.16	7.74
R1Δ1 C1	B	58	9	15.52	7.77	27.93
	S	157	23	14.65	9.70	21.38
	O	118	22	18.64	12.30	27.09
R1Δ1 C2	B	36	5	13.89	5.23	30.29
	S	58	6	10.34	4.28	21.84
	O	252	6	2.38	0.97	5.36
S9Δ2 C1	B	203	0	0.00	0.04	2.32
	S	125	1	0.80	0.04	5.03
	O	141	1	0.71	0.04	4.48
S9Δ2 C2	B	211	3	1.42	0.37	4.44
	S	408	5	1.23	0.45	3.00
	O	97	2	2.06	0.36	7.97
R1Δ2 C1	B	95	2	2.11	0.37	8.13
	S	247	7	2.83	1.25	6.00
	O	148	2	1.35	0.23	5.30
R1Δ2 C2	B	216	2	0.93	0.16	3.66
	S	235	5	2.13	0.79	5.17
	O	179	126	70.39	63.04	76.85
S9Δ4 C1	B	185	130	70.27	63.04	76.64
	S	280	58	20.71	16.22	26.03
	O	220	126	57.27	50.44	63.85
S9Δ4 C2	B	142	97	68.31	59.90	75.71
	S	191	54	28.27	22.12	35.31
	O	35	23	65.71	47.74	80.32
R1Δ4 C1	B	21	16	76.19	52.45	90.88
	S	58	17	29.31	18.46	42.91
	O	225	2	0.89	0.15	3.52
S9Δ2-4 C1	B	199	9	4.52	2.22	8.69
	S	331	14	4.23	2.42	7.16
	O	236	3	1.27	0.33	3.98
S9Δ2-4 C2	B	229	2	0.87	0.15	3.46
	S	393	12	3.05	1.66	5.42
	O	110	5	4.55	1.69	10.80
R1Δ2-4 C1	B	92	1	1.09	0.06	6.76
	S	109	3	2.75	0.71	8.43

P_l and P_u are the limits of the 95 % confidence interval. The rows O, B, and S show the results of measurements with orange light step-down, with a blue light pulse, or without stimulus (spontaneous). C1 and C2 refer to different clones of the deletion strain.
