# Supplementary material for: Identification of Archaea-specific chemotaxis proteins which interact with the flagellar apparatus
Source: BMC Microbiol. 2009 Mar 16;9:56. doi: 10.1186/1471-2180-9-56 (PMC2666748; doi:10.1186/1471-2180-9-56)
Supplement: Additional File 6 — Occurrence of che and fla genes in archaeal genomes. An exhaustive search for che and fla genes in archaeal genomes is presented and the detected orthologs listed as table (Table S2). Additionally, the method used for ortholog identification is described. [file 1471-2180-9-56-S6.pdf]

Supplementary Material - Occurrence of *che* and *fla* genes in archaeal genomes

che and *fla* genes in archaeal genomes

To have a reference for co-occurrence comparisons, an exhaustive search for orthologs of *che* and *fla* genes in all completely sequenced archaeal genomes which were published until October 2007 was done. Since homology searches using Psi-Blast were not sufficient to comprehensively identify homologs of some proteins (especially small proteins with rather low conservation like FlaC, D, E, F, and G were problematic), and did not allow the discrimination between orthologs and other homologs for other proteins (e. g. CheY and other response regulators), a combination of different methods was used for ortholog identification (see detailed description below). The resulting table of orthologs is shown in Table S2.

No *che* genes were detected in any archaeal genome without *fla* and flagellin genes. In contrast, several archaeal species contain *fla* genes and flagellins, but no *che* genes, leading to the conclusion that these species are motile, but their motility is not controlled by a *che* system. *che* genes have not been detected in any crenarchaeal genome. If *che* genes were found, there was always the whole set consisting of *cheA*, *cheB*, *cheC*, *cheD*, *cheR*, *cheW*, and *cheY* present. An exception is *Methanosarcina barkeri*, which has lost the *cheC* gene (the genomic position, where *cheC* is located in the other *Methanosarcina* species still contains remnants of the N-terminus of *cheC*). Several archaeal species contain multiple copies of various *che* genes; the front-runner is *Methanospirillum hungatei* with 35 genes classified as *che* orthologs by the used method. A noteworthy finding is a CheA-CheC fusion protein detected in the genome of *M. hungatei*.

If in a crenarchaeal genome *fla* genes were identified, there were at least one flagellin, *flaG*, *flaH*, *flaI*, and *flaJ*, and usually also *flaF* (except in *Aeropyrum pernix*) present. The *flaG* gene in the sequenced strain of *Sulfolobus solfataricus* is interrupted by a transposase, but this insertion is neither stable under laboratory conditions nor is it found in a closely related strain [1]. In euryarchaeota which possess *fla* genes there is additionally always a *flaD/E* gene present. *flaD* and *flaE* genes could not be discriminated by the applied method, so they were merged into one ortholog group. Two versions of *flaD/E* genes can be distinguished: The species of the classes Methanomicrobia and Archaeoglobi code for a version of a FlaD/E protein (referred to as FlaD/E^M in the following) with only low homology to the FlaD and FlaE proteins found in other euryarchaeal genomes [2, 3]. A special case is *Methanococcoides burtonii*

(class Methanomicrobia) that possesses both versions of the FlaD/E proteins, each in a complete *fla* gene region. This is an indication of lateral gene transfer (LGT) of a whole *fla* gene region. Such an LGT has also been proposed in a detailed study of the phylogenomics of the archaeal flagellum [3]. In genomes with FlaD/E^M (or in the case of *Methanococcoides burtonii* the genome region with FlaD/E^M), no *flaC* gene, or *flaC* domain fused to a *flaE* gene, was found. In all other euryarchaeota with *fla* genes, FlaC is either coded as separated protein, or as domain fused to an FlaD/E domain. Like the *che* genes, also the *fla* genes and flagellins are present in multiple copies in some genomes.

Methods for identification of Che and Fla orthologs

For identification of Che and Fla orthologs in archaeal genomes, a combination of homology search, genome region analysis, and cluster analysis based on pairwise similarity was applied.

First, *che* and *fla* gene regions were identified by Psi-Blast against an archaeal protein sequence database using *H. salinarum* CheD and FlaH as queries. The database contained the predicted proteins from all complete archaeal genomes available in GenBank in October 2007, except for *Halobacterium salinarum* NRC-1, which contains the same *che* and *fla* genes as *H. salinarum* R1 [4, 5]. CheD and FlaH were chosen as queries, because a former study had demonstrated, that they are highly conserved throughout all chemotactic or motile archaea, and they have no close non-orthologues homologs, which would make result evaluation difficult (unpublished data). All hits with an e-value of 10^{-8} or lower were accepted. This cut-off, however, was not critical, as there were no hits with e-values between 10^{-10} and 10^{-2} for CheD and 10^{-10} and 10^{-5} for FlaH.

Second, the genes in the neighbourhood of the identified *cheD* and *flaH* genes were examined by BlastP against the archaeal protein sequence database, and querying CDS and Pfam. Based on homology or identified domains, the genes were assigned to pools. The pools were: CheA, CheB, CheC, CheD, CheR, CheW, CheY, DUF439, FlaC, FlaD/E (FlaD and FlaE could not be distinguished), FlaF, FlaG, FlaH, FlaI, FlaJ. The examination of neighbouring genes was repeated, until on each side of the region three genes with no obvious relation to chemotaxis or flagellation were found.

Third, the pools were extended to identify homologs located apart from the main genome regions. For this, each member of a pool was used as query in a Blast search against the archaeal protein sequence database. All hits with an e-value of 10^{-3} or smaller were included into the extended pools.

Fourth, the extended pools were clustered based on pairwise similarity. This was done with the CLANS application [6]. Iteration was run until movement of vertices became negligible. The cluster in which the members of the non-extended pools were found was extracted and the members considered

as the final group of orthologs. Proteins which were not included into this ortholog cluster but did also not cluster with any other proteins and had only connections to the ortholog cluster, were included into the final ortholog group as well (marked with an asterisk in Table S2). The applicability of the method was supported by the fact that in all cases the members of the non-extended pools were found in one cluster.

Table S2: ***che* and *fla* genes in archaeal genomes.**

	**cheA**	**cheB**	**cheC**	**cheD**	**cheR**	**cheW**	**cheY**	**439**	**flaC**	**flaD/E**	**flaF**	**flaG**	**flaH**	**flaI**	**flaJ**	**flg**
Ape												1901	1898	1896.1	1895.1	1905, 1907
Afu	1040	1041	1039	1038	1037	1044	1042	1043		1053^{M}	1051	1052	1050	1049	1048	1054, 1055
Mbo	1336	1337, 0327***	1335	1334	1581, 1252, 0327***	1247, 1579, 1098	1338	1339		1346^{M}	1344	1345	1343	1342	1341	1347, 1348
Hma	2205	2204	2193, 0528, 1258, 2623	2192	2206	2203, 1484	2194	2209, 2213, 3221, 3231	2191^{F}	2191^{F}, 1482	2190	2187	2186	2184	2183	2198, pNG1026, rrnB0018*
Hsa	2415R	2416R	2410R, 2414R, 3280R	2408R	2406R	2374R, 2419R	2417R	2402F, 2404R	2386R^{F}	2390R, 2386R^{F}	2385R	2383R, 4607R	2381R	2380R	2379R	2397F, 2398F, 2399F, 2469F, 2470F, 2695F
Mse											1327	1328	1326	1325	1324	1330
Mbu	0361	0360, 0399***	0362	0363	0364, 0399***	0357	0359	0358	1570^{F}	0348^{M}, 1570^{F}, 1246	0350, 1571	0349, 1572	0351, 1573	0352, 1574	0353, 1575	0346, 0347, 0104
Mja								1615*	0894	0895, 0896	0897	0898	0899	0900	0901	0891, 0892, 0893
MC5	0734	0733	0738, 0739	0735	0737	0732	0740	0741	1738	1737, 1736	1735, 1332	1734	1733	1732	1731	1739, 1740, 1741, 1742
MC7	0174	0173	0178, 0179	0175	0177	0172	0180	0181	0942	0943, 0944	0945, 1343	0946	0947	0948	0949	0938, 0939, 0940, 0941
MS2	0927	0926	0931, 0932	0928	0930	0925	0933	0934	1669	1670, 1671	1672, 0342	1673	1674	1675	1676	1666, 1667, 1668
Mma	0943, 0238	0944, 2125**	0942	0941	1545	1544, 0240	0945	0946		0953^{M}	0951	0952	0950	0949	0948	0962, 0963, 1375, 1374

Table S2: (continued)

	**cheA**	**cheB**	**cheC**	**cheD**	**cheR**	**cheW**	**cheY**	**439**	**flaC**	**flaD/E**	**flaF**	**flaG**	**flaH**	**flaI**	**flaJ**	**flg**
Mac	3066, 0014	3067, 0015, 1989***, 3542***	3065, 0012	3064, 0011	3063, 0013, 1989***, 3542***	3070, 0020	3068, 0016	3069		3060^{M}, 3078^{M}	3058, 3080	3059, 3079	3057, 3081	3056, 3082	3055, 3083	3061, 3062, 3077
Mba	0984	0985, 2183***		0982	0983, 2183***	0990	0986, 3321			1969^{M}	1967	1968	1966	1965	1964	1970
Mmz	0328, 1325	0329, 1326	0327, 1323	0326, 1322	0325, 1324	0332, 1330	0330, 1327	0331		0321^{M}, 0417^{M}	0319, 0415	0320, 0416	0318, 0414	0317, 0413	0316, 0412	0322, 0323, 0418
Mhu	0110^{F}, 0494, 0989	0109, 0988, 0952**, 0887**	0112, 0110^{F}, 2685, 1151, 2682	0111	0961, 0992, 0124	0007, 2533, 0991, 2550, 1642, 1423, 0496, 0960, 1925, 0898, 0003, 2399, 0993*, 2532*	0108, 0126, 3040, 1439, 3041, 0315	0107		0100^{M}	0102	0101	0103	0104	0105	3140, 3139, 1238
Mva	0220	0219	0257, 0258	0221	0259	0218, 0138	0256	0255	0969	0970, 0971	0972, 1352	0973	0974	0975	0976	0966, 0967, 0968
RC-I	571	570	572, X2603	573	584	X655, X2603	569	568		512^{M}, 510^{M}	501, 500	509, 507, 506, 505, 503	499	498	497	515, 514
Mae									0261	0262, 0263	0264	0265	0266	0267	0268	0256, 0257, 0258, 0259, 0260
Nph	2172A	2174A	2104A, 3118A	2106A	2170A	4146A	2102A	2162A, 2166A	$2154A^{F}$, 2686A	$2154A^{F}$	2094A	2096A, 2098A	2156A	2158A	2160A	2086A, 2088A, 2090A
Pab	1332	1331	1334, 1333	1335	1329	1027	1330	1338	1381	1382	1383	1384	1385	1386	1387	1380, 1379, 1378*,
Pfu									0336	0335	0334	0333	0332	0331	0330	0337, 0338

Table S2: (continued)

	**cheA**	**cheB**	**cheC**	**cheD**	**cheR**	**cheW**	**cheY**	**439**	**flaC**	**flaD/E**	**flaF**	**flaG**	**flaH**	**flaI**	**flaJ**	**flg**
Pho	0484	0483	0487, 0488	0490	0481	0478	0482	0494	0552	0553	0553.1n	0555	0556	0557	0559	0546, 0548, 0549, 0550, 0551
Sac											1175	1176	1174	1173	1172	1178
Sso											2319	*is*	2318	2316	2315	2323
Sto											2521	2520	2522	2523	2524	2518
Tko	0634, 0635	0633	0636, 0637	0639	0631	0629	0632	0641	0043	0044	0045	0046	0047	0048	0049	0038, 0039, 0040, 0041, 0042
Tac									0554	0555	0556	0557a	0558	0559	0560	0553, 1407m
Tvo									0608	0609	0610	0611	0612	0613	0614	0607, 1426

The column 439 lists members of the protein family DUF439. The prefix of the gene identifiers was omitted, if the rest is unambiguous (e. g. 2415F instead of OE2415F). ^{F}: Fusion protein, belongs to two groups; ^{M}: Different version of FlaD/E protein found in Methanomicrobia and Archaeoglobi; *: singleton, not included into ortholog cluster (see text); ** protein with CheB domain but no response regulator domain; *** protein containing both a CheB and a CheR domain. *is*: gene present, but interrupted by an insertion element. The species are: Ape *Aeropyrum pernix* K1, Afu *Archaeoglobus fulgidus* DSM4304, Mbo *Candidatus Methanoregula boonei* 6A8, Hma *Haloarcula marismortui* ATCC43049, Hsa *Halobacterium salinarum* R1, Mse *Metallosphaera sedula* DSM5348, Mbu *Methanococcoides burtonii* DSM6242, Mja *Methanococcus jannaschii* DSM2661, MC5 *Methanococcus maripaludis* C5, MC7 *Methanococcus maripaludis* C7, MS2 *Methanococcus maripaludis* S2, Mma *Methanoculleus marisnigri* JR1, Mac *Methanosarcina acetivorans* C2A, Mba *Methanosarcina barkeri* fusaro, Mmz *Methanosarcina mazei* Goe1, Mhu *Methanospirillum hungatei* JF-1, Mva *Methanococcus vannielii* SB, RC-I uncultured methanogenic archaeon RC-I, Mae *Methanococcus aeolicus* Nankai-3, Nph *Natronomonas pharaonis* DSM2160, Pab *Pyrococcus abyssi* GE5, Pfu *Pyrococcus furiosus* DSM3638, Pho *Pyrococcus horikoshii* OT3, Sac *Sulfolobus acidocaldarius* DSM639, Sso *Sulfolobus solfataricus* P2, Sto *Sulfolobus tokodaii* 7, Tko *Thermococcus kodakaraensis* KOD1, Tac *Thermoplasma acidophilum* DSM1728, Tvo *Thermoplasma volcanium* GSS1. Also included in the analysis, but not listed in the table, since no *che* and *fla* orthologs were detected, were: *Haloquadratum walsbyi* DSM16790, *Hyperthermus butylicus* DSM5456, *Ignicoccus hospitalis* KIN4 I, *Methanobacterium thermoautotrophicum* delta H, *Methanobrevibacter smithii* ATCC35061, *Methanocorpusculum labreanum* Z, *Methanopyrus kandleri* AV19, *Methanosaeta thermophila* PT, *Methanosphaera stadtmanae* DSM3091, *Nanoarchaeum equitans* Kin4-M, *Picrophilus torridus* DSM9790, *Pyrobaculum aerophilum* IM2, *Pyrobaculum arsenaticum* DSM13514, *Pyrobaculum calidifontis* JCM11548, *Pyrobaculum islandicum* DSM4184, *Staphylothermus marinus* F1, *Thermofilum pendens* Hrk 5.

References

[1] Szabó Z, Sani M, Groeneveld M, Zolghadr B, Schelert J, et al. (2007) Flagellar motility and structure in the hyperthermoacidophilic archaeon *Sulfolobus solfataricus*. J Bacteriol 189:4305–4309. doi: 10.1128/JB.00042-07. URL http://dx.doi.org/10.1128/JB.00042-07.

[2] Ng SYM, Chaban B, Jarrell KF (2006) Archaeal flagella, bacterial flagella and type IV pili: a comparison of genes and posttranslational modifications. J Mol Microbiol Biotechnol 11:167–191. doi:10.1159/000094053. URL http://dx.doi.org/10.1159/000094053.

[3] Desmond E, Brochier-Armanet C, Gribaldo S (2007) Phylogenomics of the archaeal flagellum: rare horizontal gene transfer in a unique motility structure. BMC Evol Biol 7:106. doi: 10.1186/1471-2148-7-106. URL http://dx.doi.org/10.1186/1471-2148-7-106.

[4] Ng WV, Kennedy SP, Mahairas GG, Berquist B, Pan M, et al. (2000) Genome sequence of *Halobacterium* species NRC-1. Proc Natl Acad Sci U S A 97:12176–12181. doi:10.1073/pnas.190337797. URL http://dx.doi.org/10.1073/pnas.190337797.

[5] Pfeiffer F, Schuster SC, Broicher A, Falb M, Palm P, et al. (2008) Evolution in the laboratory: The genome of *Halobacterium salinarum* strain R1 compared to that of strain NRC-1. Genomics 91:335–346. doi:10.1016/j.ygeno.2008.01.001. URL http://dx.doi.org/10.1016/j.ygeno.2008.01.001.

[6] Frickey T, Lupas A (2004) CLANS: a Java application for visualizing protein families based on pairwise similarity. Bioinformatics 20:3702–3704. doi:10.1093/bioinformatics/bth444. URL http://dx.doi.org/10.1093/bioinformatics/bth444.
