# Supplementary material for: Identification of Archaea-specific chemotaxis proteins which interact with the flagellar apparatus
Source: BMC Microbiol. 2009 Mar 16;9:56. doi: 10.1186/1471-2180-9-56 (PMC2666748; doi:10.1186/1471-2180-9-56)
Supplement: Additional File 7 — Primers used in this study. This table lists the oligonucleotides used in the present study. [file 1471-2180-9-56-S7.pdf]

**Primers used in this study**

| Primer | Sequence | Restriction site |
|---|---|---|
| **Protein interaction analysis** | | |
| OE2401F_fo | CACCGTGCCATCGCTGTACGGGCTGG | |
| OE2401F_re | CGTTTTTCCGCCCAGCTTCGAGATC | |
| OE2402F_fo | CACCATGAGCGAGTCAGAGTACAAGATAG | |
| OE2402F_re | TTCCTCGTTGATCGCTTCGCTGGC | |
| OE2404R_fo | CACCATGTCGGAATCAGCAATCGCAGAC | |
| OE2404R_re | GAGGTTGACGTCCTCGATGTGTTC | |
| OE2408R_fo | CACCGTGACGATCCGCGTTGGCGTG | |
| OE2408R_re | AATTACGTGCACCTCGGCATC | |
| OE2417R_fo | CACCATGGCGAAGCAGGTCTTACTGGTC | |
| OE2417R_re | TGCGGTGAGCACGTCCGAAATAGCG | |
| OE3280R_fo | CACCATGAGTGCCACGATCGAGCTG | |
| OE3280R_re | CGAGAGGTCGTTGAGCAGCGAC | |
| **Construction of deletion mutants** | | |
| OE2401F_us_fo | ATTCGAGGATCCGGTGAGGAAGCCTCCGCACAGG | BamHI |
| OE2401F_us_re | AAACCGCCACCAGTGGCCTAGACAGACGACACGCGAC | |
| OE2401F_ds_fo | GTCGTCTGTCTAGGCCACTGGTGGCGGTTTCAGTCGC | |
| OE2401F_ds_re | TCCTATAAGCTTGCGGGATGCTTCACCTGTAGC | HindIII |
| OE2402F_us_fo | TTAACGGGATCCACACCAGCGCCGGAACGCCACG | BamHI |
| OE2402F_us_re | GGAGCAGCCCCACGATCCTCACCTACGATCGGCGCTTC | |
| OE2402F_ds_fo | GATCGTAGGTGAGGATCGTGGGGCTGCTCCCCTCGG | |
| OE2402F_ds_re | TATCGGAAGCTTCTCGCGCTCTCGCCGGGTGACC | HindIII |
| OE2404R_us_fo | TTAACGTCTAGACGTCTCCATTCTGGCCACCGAC | XbaI |
| OE2404R_us_re | TCCCCTCGGACGCCACACCCCCGTATACTCAATTATAC | |
| OE2404R_ds_fo | TGAGTATACGGGGGTGTGGCGTCCGAGGGGAGCAGCC | |
| OE2404R_ds_re | TTAAGCGGATCCGCATCAAGGTCGACGAATCGG | BamHI |
| OE2402F-OE2404R_us_re | TGAGTATACGGGGGTTCCTCACCTACGATCGGCGCTTC | |
| OE2402F-OE2404R_ds_fo | GATCGTAGGTGAGGAACCCCCGTATACTCAATTATAC | |
| **qRT-PCR** | | |
| RT-PCR_OE2381R_for | AAGTCAGCCATGAGCCAGC | |
| RT-PCR_OE2381R_rev | CCTCGACGTCATAACTCAAC | |
| RT-PCR_OE2406R_for | GTTGCTCGACACCCTCTCG | |
| RT-PCR_OE2406R_rev | CTCCAGATGTCGATCGAGC | |
| RT-PCR_OE2417R_for | TCCGGGAGATTCTTGAGGAG | |
| RT-PCR_OE2417R_rev | TGTCCATCATCACGAGATCC | |
